# Tet2-driven clonal hematopoiesis drives aortic aneurysm via macrophage-to-osteoclast–like differentiation

**DOI:** 10.1172/JCI198708

**Published:** 2026-02-25

**Authors:** Jun Yonekawa, Yoshimitsu Yura, Junmiao Luo, Katsuhiro Kato, Shuta Ikeda, Yohei Kawai, Tomoki Hattori, Ryotaro Okamoto, Mari Kizuki, Emiri Miura-Yura, Keita Horitani, Kyung-Duk Min, Takuo Emoto, Hiroshi Banno, Mikito Takefuji, Kenneth Walsh, Toyoaki Murohara

**Affiliations:** 1 Department of Cardiology, Nagoya University Graduate School of Medicine, Nagoya, Japan.; 2 Division of Vascular and Endovascular Surgery, Department of Surgery, Nagoya University Graduate School of Medicine, Nagoya, Japan.; 3 Department of Vascular Surgery, Aichi Medical University, Nagakute, Japan.; 4 Division of Diabetes, Department of Internal Medicine, Aichi Medical University School of Medicine, Nagakute, Japan.; 5 Department of Medicine II, Kansai Medical University, Osaka, Japan.; 6 Department of Cardiovascular and Renal Medicine, Hyogo Medical University, Hyogo, Japan.; 7 Division of Cardiovascular Medicine, Department of Internal Medicine, Kobe University Graduate School of Medicine, Kobe, Japan.; 8 Division of Cardiovascular Medicine, Robert M. Berne Cardiovascular Research Center, University of Virginia School of Medicine, Charlottesville, Virginia, USA.

**Keywords:** Aging, Cardiology, Vascular biology, Genetic risk factors, Hematopoietic stem cells, Macrophages

## Abstract

Aortic aneurysms are age-linked aortic dilations that progress silently and carry high mortality rates following rupture. Immune cells are recognized drivers of aneurysm pathogenesis. Clonal hematopoiesis is an age-related expansion of somatically mutated hematopoietic stem cells that reshapes immune function and contributes to diverse age-associated diseases. However, its contribution to aneurysm pathogenesis remains unclear. In this study, targeted ultradeep sequencing of patient specimens revealed a high prevalence of clonal hematopoiesis–associated mutations that correlated with faster aneurysm expansion. Thus, we modeled clonal hematopoiesis by competitively transplanting ten-eleven translocation 2–deficient (*Tet2*-deficient) bone marrow into apoliprotein E–KO (*Apoe*-KO) mice and induced aneurysms with angiotensin II. Mice with Tet2 clonal hematopoiesis developed significantly greater aortic dilation than did controls. Interestingly, *Tet2*-deficient macrophages adopted an acid phosphatase 5, tartrate resistant (ACP5^+^), osteoclast-like state and produced more matrix metalloproteinase 9 (MMP9). Both genetic and pharmacological inhibition of osteoclast-like differentiation suppressed the Tet2-mediated aneurysmal growth in vivo. Thus, Tet2-driven clonal hematopoiesis accelerated aortic aneurysm progression through MMP9-producing, osteoclast-like macrophages and therefore represents a tractable therapeutic axis.

## Abstract 2

Clonal hematopoiesis accelerates aortic aneurysm growth by driving immune cells to become osteoclast-like and destructive, revealing a potential target for therapy.

## Graphical abstract

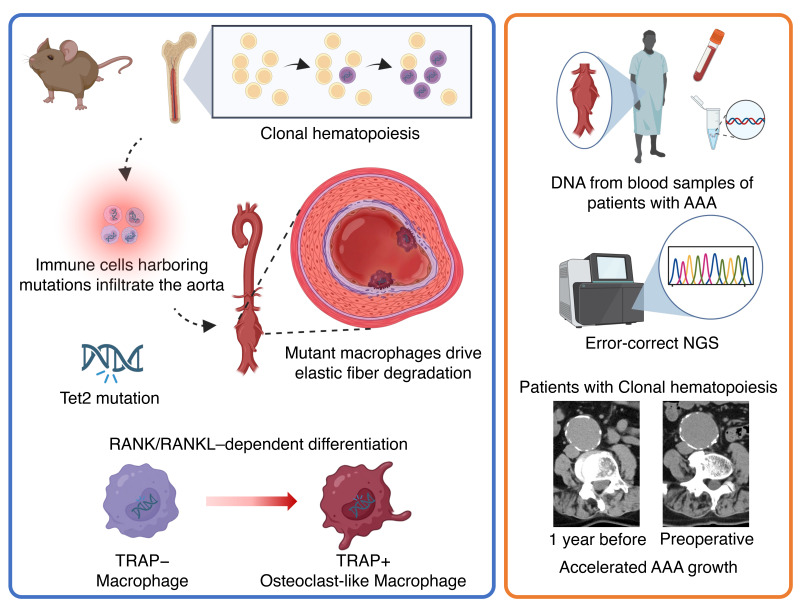

## Introduction

Aortic aneurysms have emerged as a major age-related vascular disease that progresses silently and can lead to catastrophic rupture, for which surgical intervention remains the only effective treatment (1–3). Consequently, survival rates following rupture remain low, making aortic aneurysms a major cause of sudden death. While ischemic heart disease survival rates have increased since the 1980s due to advances in the treatment of hypertension, diabetes, and dyslipidemia, as well as improvements in cardiac catheterization (4), the outcomes for aortic aneurysms remain poor, underscoring their substantial unmet medical needs (5). Moreover, the lack of dependable clinical indicators beyond aneurysm diameter to predict disease progression remains a major barrier to effective long-term management of aortic aneurysms (6). Pathological features of aortic aneurysms include extracellular matrix degradation, loss of vascular smooth muscle cells, and inflammatory cell infiltration in the aortic wall (7–9). Calcification is also observed in aneurysms, as in atherosclerosis (10). However, features such as excessive matrix degradation and smooth muscle cell loss are more pronounced in aneurysms, highlighting the importance of analyzing disease-specific immune cell involvement to better understand the mechanisms underlying aortic aneurysm.

Clonal hematopoiesis has recently drawn attention for its link to age-related diseases (11, 12). It arises from somatic mutations in hematopoietic stem and progenitor cells (HSPCs), allowing mutant clones to expand in the bone marrow (BM) (13). This can occur subclinically in older adults, often involving mutations in epigenetic regulators such as ten-eleven translocation 2 (TET2), DNA methyltransferase 3A (DNMT3A), and additional sex combs-like 1 (ASXL1), which disrupt normal hematopoiesis and immune function. As a result, immune cells derived from these mutant clones frequently adopt proinflammatory profiles, driving chronic inflammation implicated in multiple cardiovascular diseases (14, 15), as well as osteoporosis, chronic obstructive pulmonary disease, and chronic liver disease (16–18). Despite the growing body of evidence linking clonal hematopoiesis to age-related diseases, its relationship with aortic aneurysms remains largely unexplored. Therefore, this study aims to investigate the effect of clonal hematopoiesis on the development and progression of aortic aneurysms through a genetic study in humans and an experimental study in mice.

## Results

### Mice with hematopoietic Tet2 mutations exhibit augmented aortic dilatation following infusion of angiotensin II.

**Figure 1:**
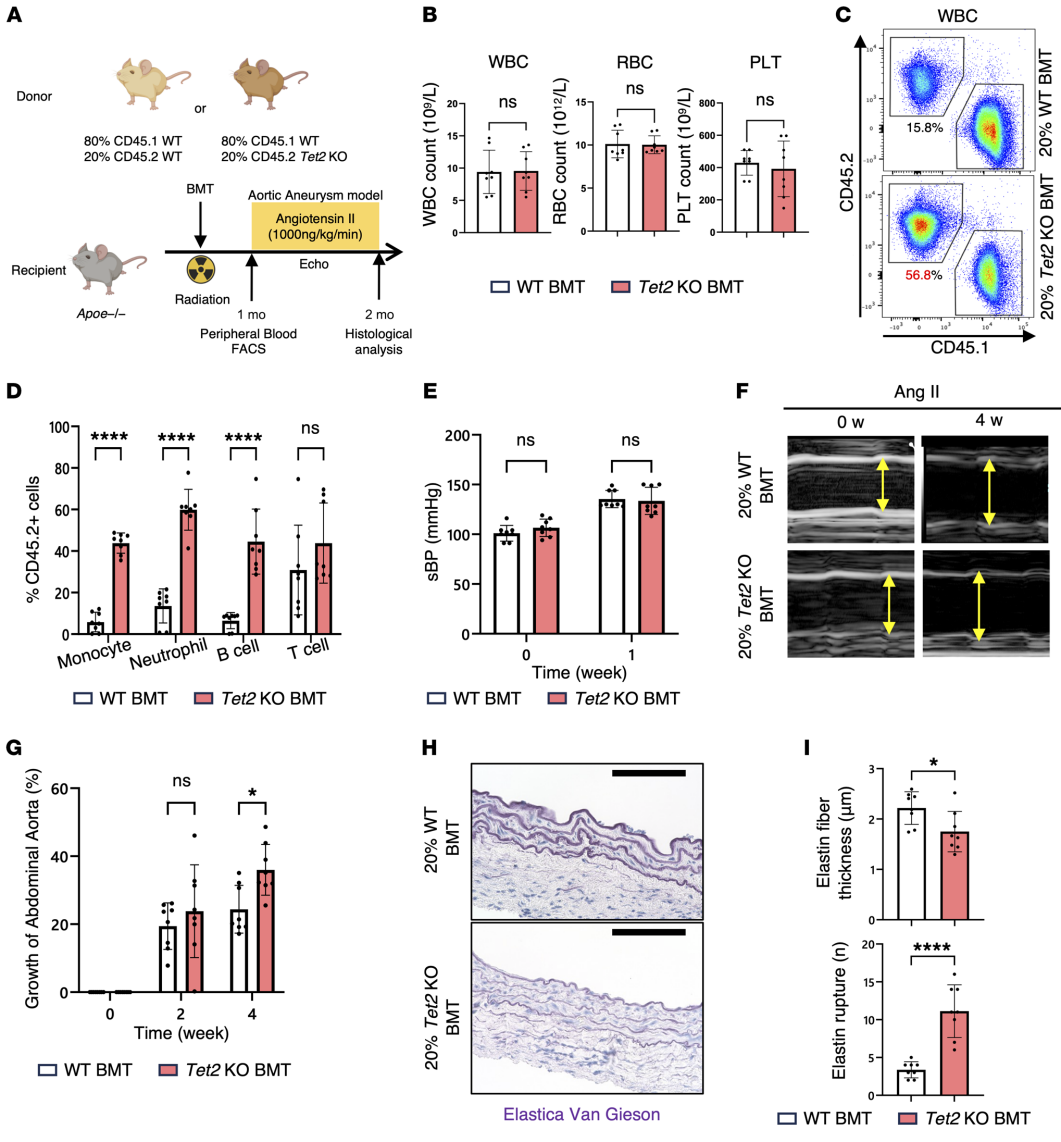
Clonal hematopoietic mouse model shows greater aortic aneurysm formation in response to AngII infusion. (**A**) Schematic of AAA model establishment using *Apo*e-KO mice and AngII infusion. To model clonal hematopoiesis, mice received 80% WT and 20% *Tet*2-KO BM cells after irradiation. Control mice received 100% WT BM. (**B**) Absolute number of WBCs, RBCs, and platelets (PLTs) in both experimental groups (*n* = 8 per genotype). Statistical significance was evaluated using an unpaired, 2-tailed Welch’s *t* test. (**C**) Representative flow cytometric gating plots of peripheral blood 4 weeks after transplantation (*n* = 8 mice per genotype). (**D**) Quantification of peripheral blood cell populations 4 weeks after transplantation (*n* = 8 mice per genotype). ****P < 0.0001, by Mann-Whitney *U* test. (**E**) Systolic blood pressure (sBP) was measured using tail-cuff plethysmography after 1 week of AngII infusion (*n* = 8 mice per genotype). *P < 0.05, by 2-way, repeated-measures ANOVA with Šidák’s multiple-comparison test. (**F**) Representative ultrasound images of the abdominal aorta at 0 (baseline) and 4 weeks after AngII infusion. Images are representative of 8 mice per genotype. (**G**) Quantification of abdominal aortic diameter at 0 (baseline), 2, and 4 weeks after AngII infusion (*n* = 8 mice per genotype). *P < 0.05, by 2-way, repeated-measures ANOVA with Šidák’s multiple-comparison test. (**H**) Representative images of Elastica van Gieson staining of abdominal aortic tissue. Scale bars: 100 μm. (**I**) Elastin fiber thickness and rupture counts in **H** (*n* = 8 mice per genotype). *P < 0.05 and ****P < 0.0001, by 2-tailed, unpaired Student’s *t* test, and for rupture counts using a 2-tailed Mann-Whitney *U* test.

To investigate the effect of clonal hematopoiesis on aortic aneurysm, we established a combined model of abdominal aortic aneurysm (AAA) and clonal hematopoiesis in mice. In human clonal hematopoiesis, a small fraction of mutant cells coexists with normal cells and gradually expands over time. To recapitulate this phenomenon in a mouse model, chimeric BM was prepared by mixing 20% mutant cells with 80% normal cells and was transplanted into myeloablated apolipoprotein E–deficient (*Apoe*-deficient) mice (Figure 1A), enabling the establishment of a clonal hematopoiesis model in which mutant cells were initially present at a low proportion. The gene was used as a representative model of clonal hematopoiesis because it is among the most biologically well-characterized clonal hematopoiesis drivers with established relevance to vascular inflammation (11). To distinguish mutant from WT cells, different CD45 isoforms were used: mutant cells were labeled with CD45.2, whereas normal cells were labeled with CD45.1. The proportion of mutant cells was then evaluated through flow cytometric analysis of peripheral blood. Despite normal peripheral blood cell counts (Figure 1B), *Tet2*-mutant cells exhibited robust expansion in the peripheral blood (Figure 1, C and D), consistent with the clinical paradigm of clonal hematopoiesis. At 1 month after bone marrow transplantation (BMT), echocardiographic assessment revealed no overt abnormalities in aortic structure, and there were no differences in aortic diameter or blood pressure between mice receiving control cells versus those receiving *Tet2*-mutant cells. These results indicate that, at baseline, mice harboring a substantial fraction of *Tet2*-mutant HSPCs did not exhibit overt hematologic or vascular abnormalities.

The model described above was then subjected to angiotensin II (AngII) stimulation, a well-established method for inducing AAA (19). Both *Tet2*-mutant (*Tet2^–/–^*) and WT (*Tet2^+/+^*) groups showed comparable increases in systolic blood pressure (Figure 1E). Nevertheless, echocardiographic analyses revealed a marked dilation of the abdominal aorta in the hematopoietic *Tet2*-mutant mice (Figure 1, F and G). Histological analysis confirmed thinning and fragmentation of elastic fibers in the vascular media of *Tet2*-mutated mice (Figure 1, H and I). Collectively, these findings demonstrate that Tet2-driven clonal hematopoiesis accelerates aortic expansion, underscoring its pathogenic role in AAA.

### Detection of clonal hematopoiesis in patients with aortic aneurysm.

**Figure 2:**
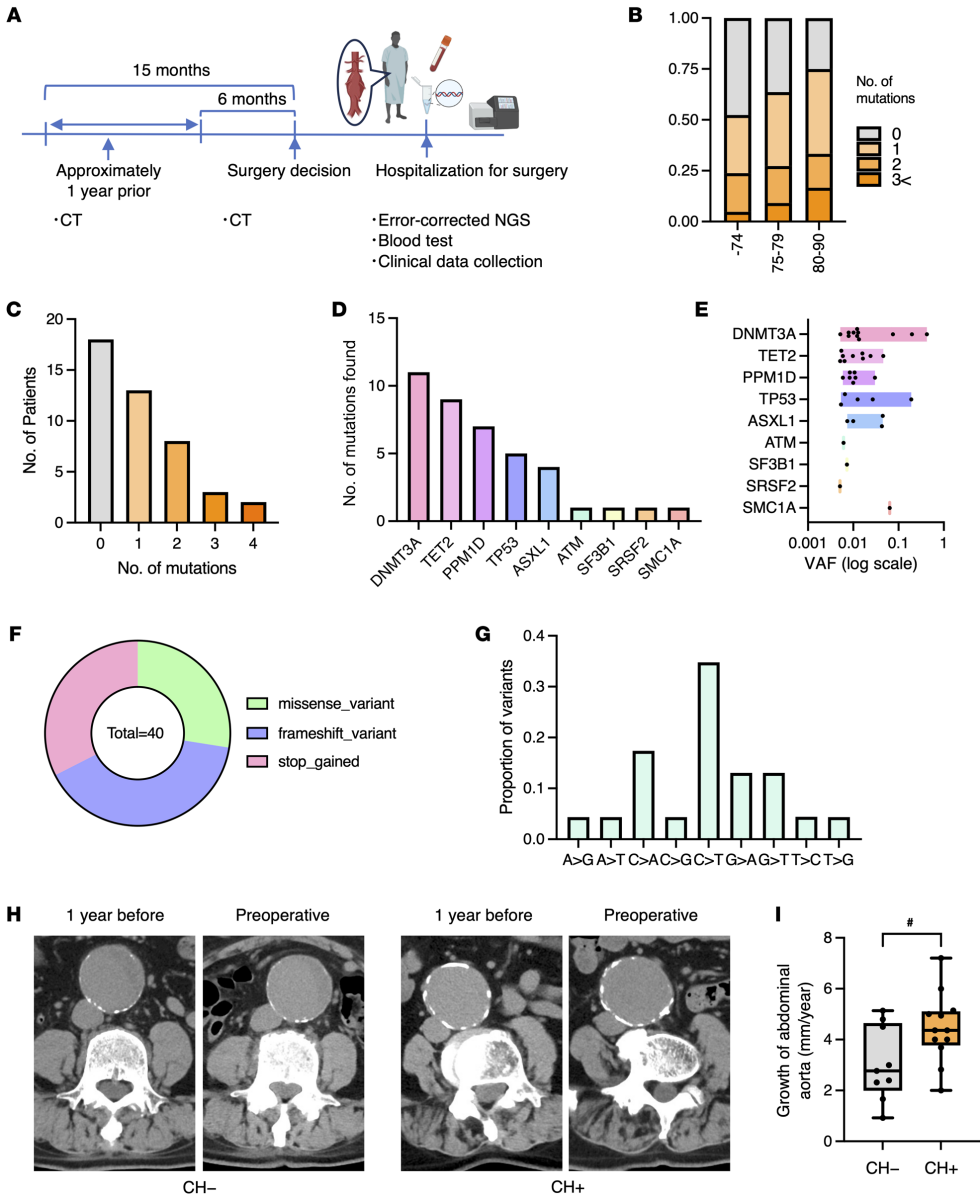
Analysis of clonal hematopoiesis in patients with aortic aneurysm. (**A**) Schematic overview of the clinical follow-up protocol. (**B**) Age-stratified prevalence of the number of mutations per individual. (**C**) Proportion of patients with the specified number of clonal hematopoiesis mutations (*n* = 44). (**D**) Abundance of the specified driver gene mutations (*n* = 40). (**E**) VAF distribution of driver genes (*n* = 40). The *x* axis is shown on a logarithmic scale. (**F**) Proportions of different types of gene mutations (*n* = 40). (**G**) Proportions of different nucleotide substitutions (*n* = 24). (**H**) Representative CT images of aortic aneurysms with and without clonal hematopoiesis (CH). Images are representative of 12 patients with clonal hematopoiesis and 9 patients without clonal hematopoiesis. (**I**) Aortic expansion rate in patients with clonal hematopoiesis (*n* = 12) and those without (*n* = 9). Data are presented as the mean ± SEM. ^#^*P* = 0.036, by unpaired, 2-tailed Welch’s *t* test.

To assess the clinical relevance of our findings, we analyzed peripheral blood samples from 44 patients with aortic aneurysm undergoing stent graft placement (Figure 2A). Using error-corrected next-generation sequencing (NGS), we quantified clonal hematopoiesis-related mutations in DNA extracted from blood samples collected from these patients (20). Targeted analysis included 17 genes, including those involved in epigenetic regulation (DNMT3A, TET2, and ASXL1), DNA damage response pathways (tumor protein p53 [TP53], protein phosphatase, Mg^2+^/Mn^2+^ dependent 1D [PPM1D], ataxia telangiectasia mutated [ATM], and checkpoint kinase 2 [CHK2]), and RNA splicing (serine and arginine rich splicing factor 2 [SRSF2] and splicing factor 3b subunit 1 [SF3B1]). The full list is shown in Supplemental Table 1; supplemental material available online with this article; https://doi.org/10.1172/JCI198708DS1 The presence of these mutations and the proportion of mutated cells were quantified. As expected, we observed an increase in the proportion of individuals with clonal hematopoiesis in older group when dividing patients into 3 age groups (<74 years old, 75–79 years old, 80–90 years old) (Figure 2B). Collectively, we found that 60% of patients carried at least 1 gene mutation, and 35% harbored 2 or more (Figure 2C). Notably, the overall frequency of clonal hematopoiesis–related mutations, particularly in DNMT3A and TET2 (Figure 2D), was higher than the prevalence reported in population-based cohorts using non-error-corrected sequencing (21). The majority of the variant allele frequencies (VAFs) ranged from 0.5% to 2%, although larger clones exceeding 10% were also observed in some cases (Figure 2E). In terms of mutation types, missense, frameshift, and stop codon gain mutations were observed at roughly equal frequencies (Figure 2F). Numerous amino acid substitutions were identified, and C>T substitutions in DNA were the most common (Figure 2G).

Table 1 summarizes the baseline characteristics of the study population. When patients with clonal hematopoiesis were compared with those without clonal hematopoiesis, no statistically significant differences were observed in age (75.8 ± 6.5 vs 73.4 ± 8.7 years, *P* = 0.33), sex (male: 89.3 % vs 93.7 %, *P* = 0.63), BMI, estimated glomerular filtration rate (eGFR), or the prevalence of hypertension, diabetes mellitus, dyslipidemia, coronary artery disease, heart failure, smoking status, family history, or surgical method. Next, we retrospectively analyzed the expansion of aortic aneurysms in patients for whom longitudinal imaging was available. Table 2 shows the characteristics of this subgroup, which was comparable to the overall cohort with regard to demographic and clinical characteristics. Follow-up duration was similar between the negative and positive clonal hematopoiesis groups (no clonal hematopoiesis: 11.4 ± 2.6 months; clonal hematopoiesis: 11.3 ± 2.7 months, respectively). Representative CT scan images from patients with and without clonal hematopoiesis, taken 1 year before intervention and just prior to intervention, are shown (Figure 2H). Strikingly, patients with clonal hematopoiesis exhibited significantly greater aortic aneurysm growth prior to requiring surgical intervention (4.3 ± 2.8 vs. 2.8 ± 1.3 mm/year, *P* = 0.036) (Figure 2I). We assessed whether clone size at surgery was associated with aneurysm progression and found no association between VAF and aneurysm growth rate in univariable linear regression analysis. These clinical data support our experimental findings and suggest that clonal hematopoiesis contributes to aortic aneurysm progression in humans.

### BM-derived macrophages play an essential role in aortic dilatation driven by Tet2 clonal hematopoiesis.

**Figure 3:**
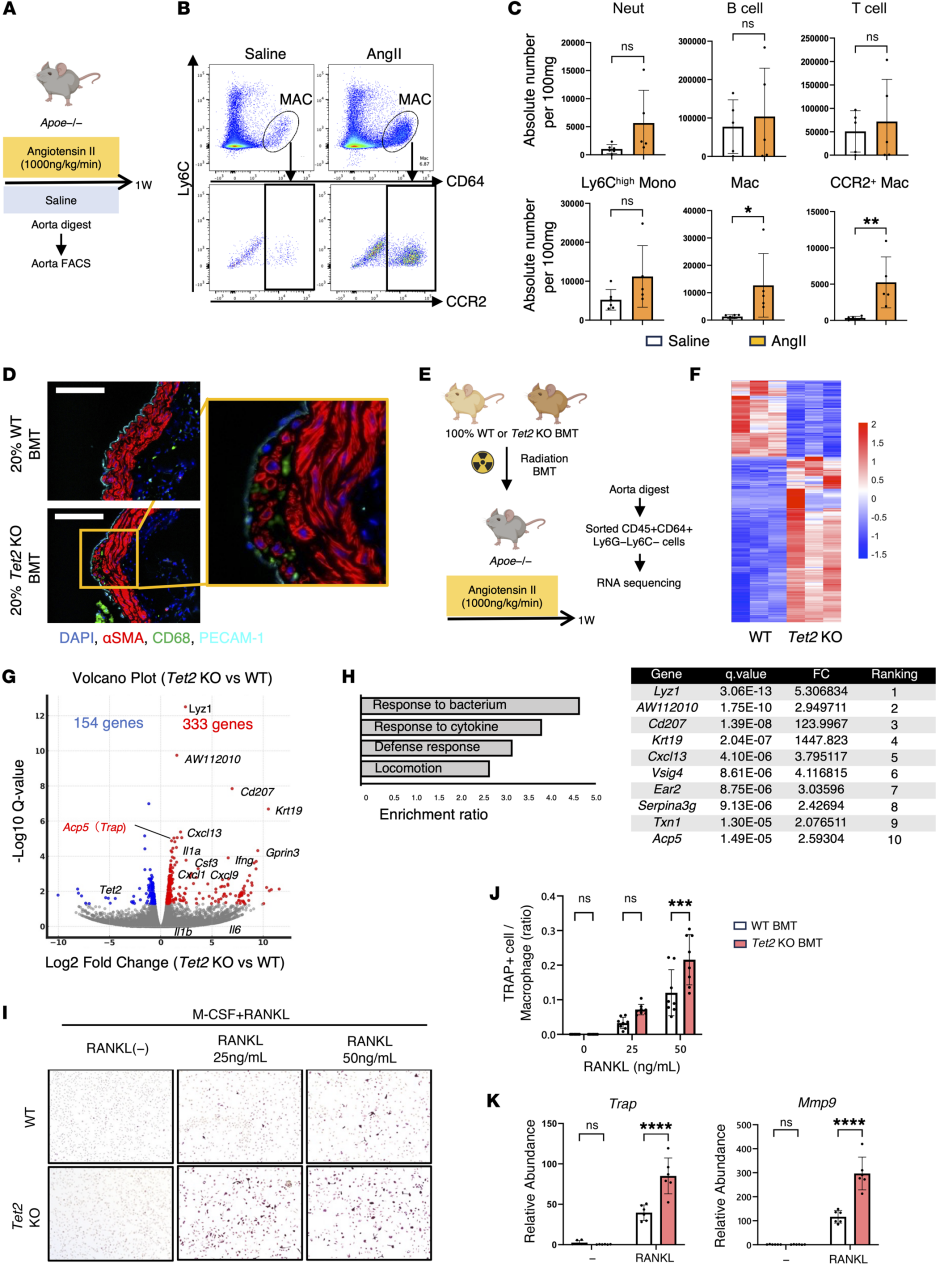
BM-derived macrophage involvement in experimental aortic aneurysm. (**A**) Schematic of flow cytometric analysis and quantification of immune cells in abdominal aortic tissue from the AAA model after 1 week of AngII infusion. (**B**) Representative flow cytometry plots of digested abdominal aortic tissue 1 week after AngII infusion (*n* = 5 mice per group). (**C**) Quantification of immune cell populations in digested abdominal aortic tissue (absolute number per 100 mg). Neut, neutrophils; Ly6C^hi^ Mono, Ly6C^hi^ monocytes; Mac, macrophages; CCR2^+^ Mac, CCR2^+^ macrophages. Saline, *n* = 6; AngII, *n* = 5; B cells and T cells, *n* = 5 per group. *P < 0.05 and **P < 0.01, by Mann-Whitney *U* test. (**D**) Representative images of CD68, α–smooth muscle actin (α-SMA), CD31/platelet endothelial cell adhesion molecule 1 (PECAM-1), and DAPI immunofluorescence staining of abdominal aortic tissue from aortas of control and 20% *Tet2*-KO BM recipient mice after 1 week of AngII infusion. Images are representative of 8 mice per genotype. Scale bars: 100 μm. (**E**) Schematic of RNA-seq analysis of sorted macrophages from abdominal aortas of 100% *Tet2*-KO BM recipient mice after 1 week of AngII infusion. (**F** and **G**) Upregulated and downregulated genes are presented as a heatmap (**F**) and volcano plot (**G**). (**H**) Gene Ontology enrichment analysis of upregulated genes in *Tet2*-deficient macrophages. The table lists the predominant genes ranked by *q* value among significantly enriched pathways. (**I** and **J**) In vitro differentiation of BM-derived macrophages. (**I** and **J**) Representative images and quantification of the TRAP^+^ cell-to-macrophage ratio after RANKL stimulation from 8 independent biological replicates per genotype. ***P < 0.001, by Mann-Whitney *U* test. (**K**) qRT-PCR analysis of BM-derived macrophages 6 hours after stimulation with 10 ng/mL LPS (*n* = 6 independent biological replicates per genotype). ****P < 0.0001, by Mann-Whitney *U* test.

Somatic mutations that arise in hematopoietic stem cells are passed on to all multiple blood lineages. Thus, to clarify the culprit cell population in AAA progression in the mouse clonal hematopoiesis model, we infused AngII into *Apoe*-deficient mice and profiled aortic immune cells by flow cytometry 1 week later (Figure 3A). Among the major leukocyte subsets, including monocytes, neutrophils, macrophages, T cells, and B cells, only macrophages were found to be significantly increased in the AngII condition relative to the saline control, although there were nonsignificant trends toward increased numbers of neutrophils and Ly6C^hi^ monocytes (Figure 3, B and C, and Supplemental Figure 1). We further distinguished yolk sac–derived CCR2^–^ macrophages from BM-derived CCR2^+^ macrophages and observed a selective expansion of the CCR2^+^ subset (Figure 3, B and C). Collectively, these findings indicate that BM-derived macrophages may participate in the accelerated development of AAA.

Immunostaining of aortic tissue in *Tet2*-mutant mice revealed that macrophages accumulated beneath the vascular endothelium, coinciding with abnormal smooth muscle cell proliferation in the aortic wall (Figure 3D). To further investigate their role, we isolated macrophages from aorta using FACS and subjected them to RNA-seq (Figure 3E). This analysis identified 333 significantly upregulated genes and 154 downregulated genes (Figure 3, F–H) in the *Tet2*-mutant condition. Among the differentially expressed genes, *Acp5* (tartrate-resistant acid phosphatase type 5 [TRAP]) was among the genes with the most significant *q* values, which was an unexpected finding. Given prior reports implicating TRAP^+^ macrophages in aneurysm pathology (22), we focused subsequent analyses on TRAP during osteoclast-like cell differentiation. TRAP^+^ macrophages are known to secrete MMP9, an enzyme that degrades elastic fibers and contributes to aneurysm progression (22). Consistent with the RNA-seq data from aortic cells, cultured BM-derived *Tet2*-mutant macrophages showed an increased propensity to differentiate into TRAP^+^ cells and had elevated *Mmp9* mRNA expression (Figure 3, I–K). Therefore, we hypothesized that the underlying mechanism could involve the propensity of *Tet2*-mutant macrophages to differentiate into TRAP^+^ macrophages, leading to MMP9 production and the destruction of elastic fibers that contribute to aortic aneurysm expansion.

### The RANKL pathway has a critical role in TRAP^+^ macrophage differentiation and AAA disease progression in mice with Tet2 clonal hematopoiesis.

**Figure 4:**
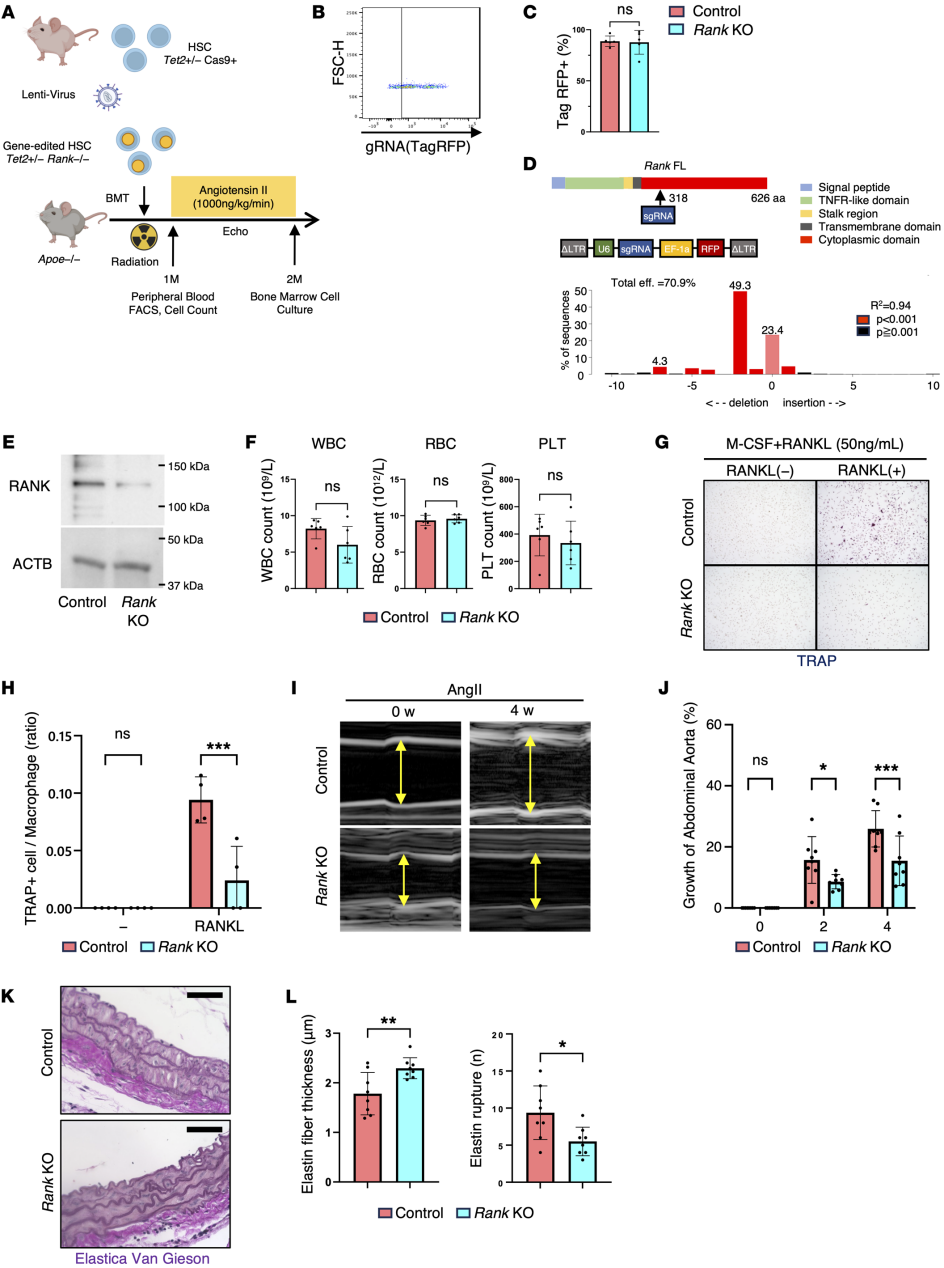
The RANK/RANKL pathway has critical role in TRAP^+^ macrophage differentiation and AAA disease progression in mice with clonal hematopoiesis. (**A**) Schematic of *Tet2*/*Rank* double-KO model. BM-derived lineage-negative cells from Cas9-expressing *Tet2^+/–^* mice were transduced with lentivirus particles expressing sgRNA/TagRFP and delivered to lethally irradiated *Apoe*-KO mice. One month after BMT, peripheral blood was collected for flow cytometric analysis. (**B**) Expression of sgRNA/TagRFP vector at 4 weeks after BMT in CD45^+^ cells. FSC-H, forward scatter height. (**C**) Quantitative analysis of TagRFP^+^ expression in cell populations derived from HSPCs transduced with lentiviral vectors encoding control or RANK-targeting sgRNA (*n* = 5 mice per genotype). Statistical significance was evaluated using Mann-Whitney *U* test. (**D**) Tracking of indels by decomposition analysis of the TagRFP^+^ peripheral blood cells revealing insertions and deletions. (**E**) Immunoblot analysis of RANK expression in BM-derived macrophages. Anti-RANK antibody detected multiple immunoreactive bands; the approximately 100–150 kDa band (putative modified form of RANK) is shown for KO validation. (**F**) Absolute number of WBCs, RBCs, and PLTs in both experimental groups (*n* = 6 mice per genotype). Statistical significance was evaluated using an unpaired, 2-tailed Welch’s *t* test. (**G** and **H**) In vitro differentiation of BM-derived macrophages. Representative images and quantification of the TRAP^+^ cell-to-macrophage ratio after RANKL stimulation from 4 independent biological replicates per genotype. Original magnification, ×10. ***P < 0.001, by Mann-Whitney *U* test. (**I**) Representative ultrasound images of the abdominal aorta at 0 (baseline) and 4 weeks after AngII infusion (*n* = 8 mice per genotype). (**J**) Quantification of abdominal aortic diameter at 0 (baseline), 2, and after 4 weeks of AngII infusion in control and RANK-KO mice (*n* = 8 mice per genotype). *P < 0.05 and ***P < 0.001, by 2-way, repeated-measures ANOVA with Šidák’s multiple-comparison test. (**K**) Representative images of Elastica van Gieson staining of abdominal aortic tissue. Scale bars: 100 μm. (**L**) Elastin fiber thickness and rupture counts in **J** (*n* = 8 mice per genotype). *P < 0.0 and **P < 0.01. Statistical significance for elastin fiber thickness was evaluated using a 2-tailed, unpaired Student’s *t* test, and for rupture counts using a 2-tailed Mann-Whitney *U* test.

To directly test the role of TRAP^+^ cells in *Tet2*-mediated acceleration of AAA, we disrupted the RANK/RANKL pathway, which plays a key role in promoting macrophage differentiation into osteoclast-like TRAP^+^ cells (23, 24). RANK is a type I transmembrane receptor composed of an extracellular domain containing multiple cysteine-rich TNF receptor–like motifs, a stalk region, a transmembrane domain, and a cytoplasmic tail that recruits adaptor proteins such as TRAF6 to activate downstream pathways including NF-κB (23, 25). To achieve a functional KO of RANK, we used a CRISPR/Cas9 strategy targeting the cytoplasmic domain of murine *Rank* and introduced a frameshift mutation predicted to disrupt downstream signaling. BM cells were harvested from *Tet2^+/–^* Cas9-expressing mice, enriched for hematopoietic stem cells by removal of lineage-positive cells, and then infected with lentiviruses expressing murine *Rank*–targeting gRNA and the reporter protein RFP to induce CRISPR/Cas9-mediated gene editing prior to transplantation (Figure 4A). One month after BMT, we collected peripheral blood and performed flow cytometry. This analysis revealed that approximately 90% of CD45^+^ cells were positive for red fluorescent protein (RFP^+^), indicating successful lentivirus infection (Figure 4, B and C). Additionally, insertion and deletion (Indel) mutations were observed in 70% of the CD45 cells, verifying efficient gene editing (Figure 4D). Western blot analysis using a RANK antibody recognizing the N-terminal extracellular domain confirmed the loss of RANK protein expression in edited cells (Figure 4E). Importantly, these mice with the edited *Rank* gene exhibited no overt abnormalities in peripheral blood cell counts (Figure 4F). Using BM cells from these mice, an in vitro osteoclast differentiation assay demonstrated that RANK deficiency significantly suppressed macrophage differentiation into TRAP^+^ cells after RANKL treatment (Figure 4, G and H). We next administered AngII to evaluate aortic dilation in the clonal hematopoiesis/AAA model. Compared with *Tet2*-mutant *Rank* WT mice, mice transplanted with *Tet2*-*Rank* double-mutant BM exhibited significantly greater reductions in aortic aneurysm expansion, thicker elastin fibers, and less fiber rupture (Figure 4, I–L). Collectively, these findings demonstrate that RANK deficiency suppressed macrophage differentiation into osteoclast-like TRAP^+^ cells and ameliorated the aortic aneurysms observed in the Tet2 clonal hematopoiesis models, underscoring the critical role of this pathway in disease progression.

### Pharmacological inhibition of macrophage differentiation into TRAP^+^cells ameliorates AAA in mice with Tet2 clonal hematopoiesis.

**Figure 5:**
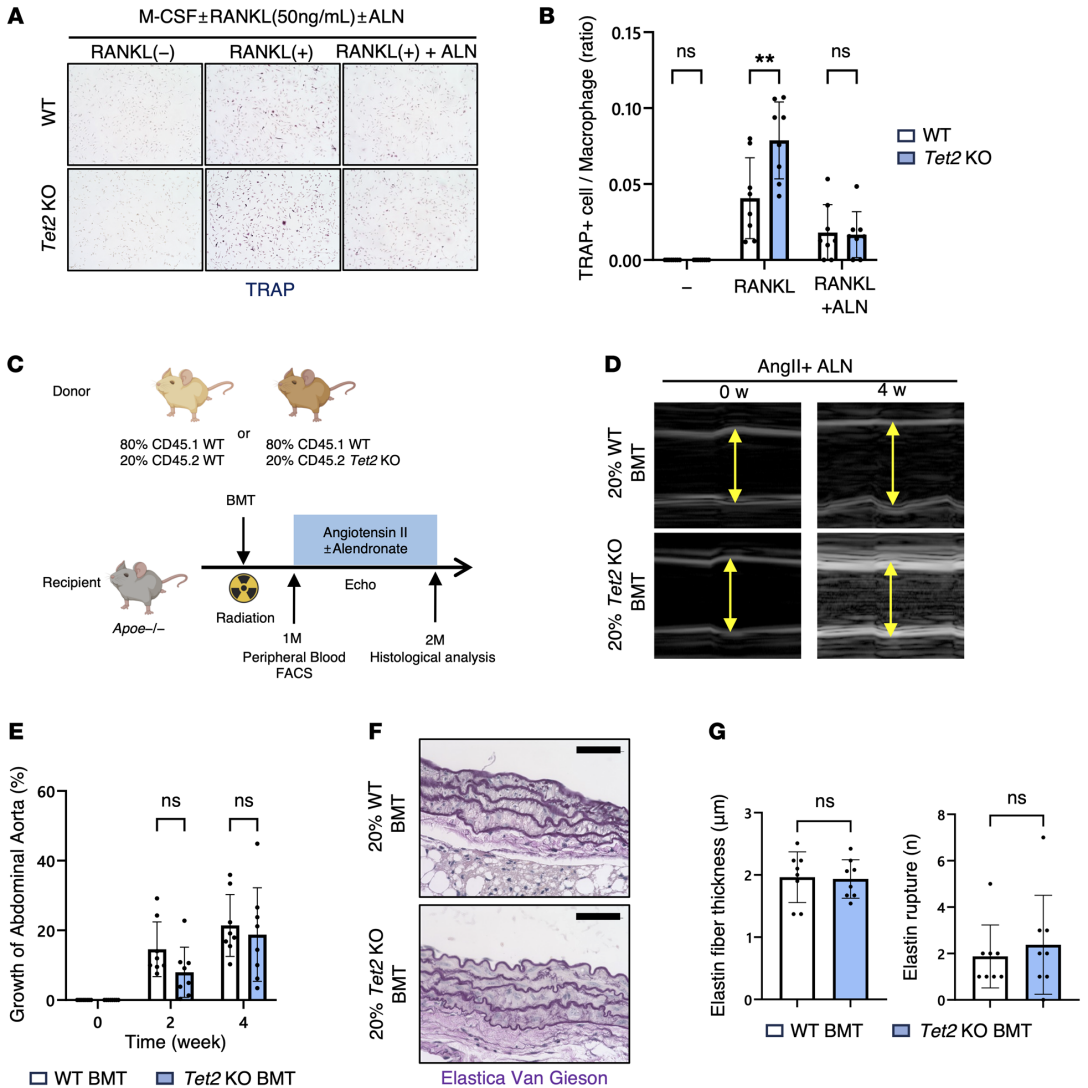
Pharmacological inhibition of TRAP^+^ macrophage ameliorates AAA phenotype in mice with Tet2 clonal hematopoiesis. (**A** and **B**) In vitro differentiation of BM-derived macrophages. Representative images and quantification of the TRAP^+^ cell-to-macrophage ratio after RANKL stimulation, with or without alendronate (ALN) treatment (*n* = 8 independent biological replicates per genotype). Original magnification, ×10. **P < 0.01, by Mann-Whitney *U* test. (**C**) Schematic of in vivo experimental design using the clonal hematopoiesis and AAA model with additional alendronate treatment. (**D**) Representative ultrasound images of the abdominal aorta at 0 (baseline) and 4 weeks after AngII infusion (*n* = 8 mice per genotype). (**E**) Quantification of the abdominal aortic diameter at 0 (baseline), 2, and 4 weeks after AngII infusion with alendronate treatment (*n* = 8 mice per genotype). Statistical significance was evaluated using 2-way, repeated-measures ANOVA with Šidák’s multiple-comparison test. (**F** and **G**) Representative images of Elastica van Gieson staining of abdominal aortic tissue and quantification of elastin fiber thickness and elastin rupture (*n* = 8 mice per genotype. Scale bars: 100 μm. Statistical significance for elastin fiber thickness was evaluated using a 2-tailed, unpaired Student’s *t* test, and for rupture counts using a 2-tailed Mann-Whitney *U* test.

To corroborate these genetic findings, we investigated the efficacy of a pharmacological inhibitor targeting osteoclasts. Bisphosphonates, such as alendronate, inhibit the mevalonate pathway in osteoclast precursors by blocking the prenylation of small GTPase proteins (26). Through this mechanism, alendronate prevents the differentiation of macrophages into TRAP^+^ osteoclast-like cells. Thus, cell culture experiments were performed to test whether this drug could suppress the differentiation of *Tet2*-mutant macrophages into TRAP^+^ osteoclast-like cells. *Tet2*-mutant macrophages, which exhibit an increased propensity for osteoclast-like differentiation, were significantly inhibited in their differentiation into TRAP^+^ cells upon treatment with alendronate (Figure 5, A and B). To determine whether pharmacological inhibition of this pathway could ameliorate the aortic aneurysm phenotype, AAA model mice were also treated with alendronate via s.c. injection (Figure 5C). Alendronate significantly ameliorated the phenotype in the angiotensin-induced AAA model mice, and the differences between the WT and Tet2 groups were no longer statistically significant (Figure 5, D–G). In addition to the s.c. injection, oral alendronate was administered daily by gavage after a 4-hour fast, with food withheld for 30 minutes after the injection, beginning on day 0 of AngII infusion (Supplemental Figure 3A). Similar to the s.c. treatment, we found that oral alendronate markedly suppressed aortic aneurysm expansion and that the difference between *Tet2*-mutant and WT groups was no longer detectable (Supplemental Figure 3, B–E), indicating that the more clinically relevant route of oral alendronate administration was also effective in attenuating aneurysm progression.

**Figure 6:**
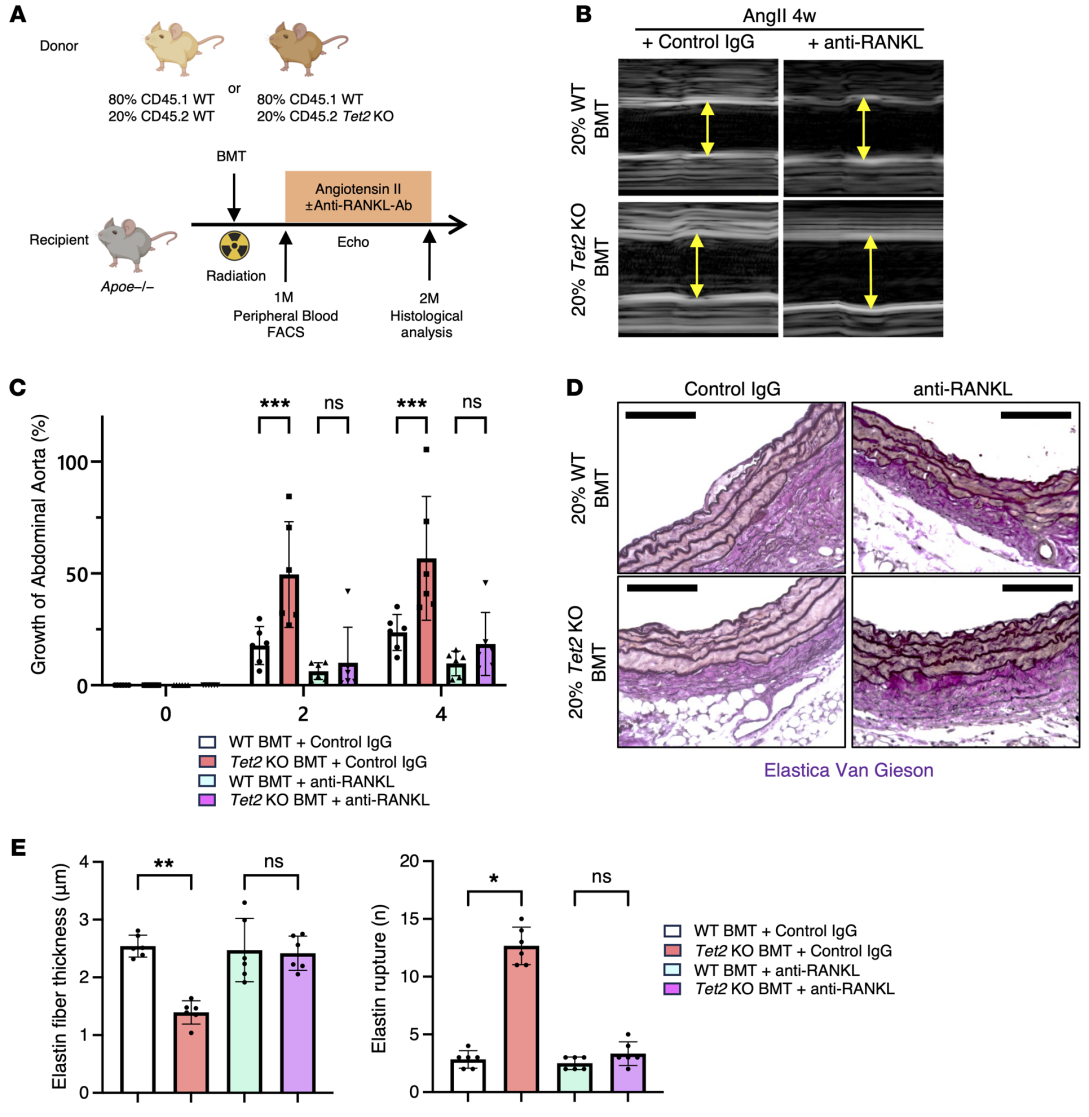
Pharmacological inhibition of TRAP^+^ macrophages by anti-RANKL antibody ameliorates AAA phenotype in mice with clonal hematopoiesis. (**A**) Schematic of in vivo experimental design using clonal hematopoiesis and AAA model with anti-RANKL antibody treatment. (**B**) Representative ultrasound images of the abdominal aorta 4 weeks after AngII infusion (*n* = 6 mice per group). (**C**) Quantification of the abdominal aortic diameter at 0 (baseline), 2, and 4 weeks after AngII infusion with anti-RANKL antibody treatment (*n* = 6 mice per group). ***P < 0.001, by 2-way, repeated-measures ANOVA with Šidák’s multiple-comparison test. (**D** and **E**) Representative Elastica van Gieson staining images of abdominal aortic tissue and quantification of elastin fiber thickness and elastin rupture (*n* = 6 mice per group). Scale bars: 100 μm. *P < 0.05 and **P < 0.01, by Kruskal-Wallis test followed by Dunn’s multiple-comparison test. Selected pairwise comparisons are shown for clarity.

Finally, a separate cohort of mice received once every 2 days an anti-RANKL monoclonal antibody that prevents osteoclast-like differentiation of macrophages (27, 28) (Figure 6A). As expected, the control IgG had no effect on aneurysm formation, as the *Tet2*-mutant group treated with control IgG exhibited accelerated aneurysm expansion (Figure 6, B and C), consistent with our initial experiments (Figure 1, F and G). Strikingly, mice treated with anti-RANKL antibody had significantly reduced aortic diameters compared with IgG-treated controls in both the WT and *Tet2*-mutant groups, and the difference between genotypes became undetectable (Figure 6, B–E). Collectively, these findings further support a causal role for RANK/RANKL/TRAP-mediated macrophage differentiation in promoting extracellular matrix degradation in *Tet2*-mutant conditions.

## Discussion

Aortic aneurysm is a life-threatening vascular condition that often progresses silently and can result in fatal rupture (1). Despite the recognition of classical cardiovascular risk factors such as hypertension and dyslipidemia in aortic aneurysm pathogenesis, current pharmacological interventions targeting these factors have had limited efficacy in preventing aneurysm progression (5). Furthermore, no biomarkers have been identified that stratify patients by risk of aneurysm progression (6). These features highlight the unmet medical need in aortic aneurysm management and suggest that previously unrecognized mechanisms contribute to its pathology.

Studies have implicated various immune cells as key mediators of aortic aneurysm pathogenesis (7). Clonal hematopoiesis, a condition characterized by the age-related expansion of hematopoietic stem cells carrying somatic mutations, has emerged as a novel contributor to age-associated diseases by altering immune cell function (11). We therefore hypothesized that clonal hematopoiesis accelerates aortic aneurysm progression and set out to test this hypothesis experimentally and clinically. To determine whether clonal hematopoiesis is associated with aortic aneurysm progression in humans, we performed ultra-deep, error-corrected sequencing of blood samples from patients undergoing surgical repair for aortic aneurysms. We observed a high prevalence of mutations in clonal hematopoiesis–related genes, including *TET2* and *DNMT3A*. Notably, patients with detectable clonal hematopoiesis exhibited more rapid aneurysm expansion prior to intervention, supporting a potential clinical link between these conditions.

Among known clonal hematopoiesis driver mutations, TET2 is of particular interest due to its known effects on myeloid cell activation and cytokine production (20, 29–31). To test whether Tet2 clonal hematopoiesis can affect aortic aneurysms in an experimental system, we established a competitive BMT model in *Apoe*-KO mice using BM cells that showed the expansion of the mutant hematopoietic cells. Upon AngII infusion, hematopoietic *Tet2*-mutant mice developed markedly larger aortic aneurysms than did controls, along with marked elastin degradation and macrophage infiltration. Taken together, these mouse and human data identify clonal hematopoiesis as a previously unrecognized candidate accelerator of aortic aneurysms, with *Tet2* mutations mechanistically validated in a mouse model. Furthermore, flow cytometric and transcriptomics analyses revealed the selective expansion of CCR2^+^ *Tet2*-mutant macrophages with features suggestive of osteoclast-like differentiation. These findings raised the possibility that the *Tet2* mutation alters myeloid cell fate and contributes to aneurysm progression.

Mechanistically, we found that *Tet2*-deficient macrophages in the aortic wall displayed transcriptional signatures indicative of osteoclast differentiation. RNA-seq of FACS-sorted macrophages revealed elevated expression of *Acp5* (encoding TRAP), cathepsin K (*Ctsk*), and other genes associated with bone resorption, suggesting that hematopoietic *Tet2* deficiency promoted the differentiation of macrophage into osteoclast-like cells. This phenotype was further confirmed in vitro, where *Tet2*-mutant, BM-derived macrophages exhibited enhanced RANKL-induced differentiation into TRAP^+^ multinucleated osteoclast-like cells. *Tet2*-deficient macrophages have been shown to upregulate inflammatory gene expression and produce large amounts of interleukins following stimulation (29, 32, 33). It is also known that the differentiation of macrophages into osteoclast-like cells is promoted by inflammatory conditions (34, 35). These cells also secrete higher levels of MMP9, an enzyme known to degrade extracellular matrix components and weaken the aortic wall (36–38). Thus, to validate the functional relevance of this pathway, we genetically ablated RANK or pharmacologically inhibited osteoclast function using bisphosphonates or an anti-RANKL antibody. Both approaches markedly attenuated aneurysm formation, such that the differences in aneurysm expansion between *Tet2*-mutant and WT conditions were no longer detectable. Taken together, these findings demonstrate that Tet2-driven clonal hematopoiesis promoted AAA progression by redirecting macrophage differentiation toward a tissue-destructive, osteoclast-like fate, thus providing a mechanistic link between somatic mutations in hematopoietic cells and vascular degeneration. These findings provide a rationale for future clinical investigations. Although large-scale clinical studies examining bisphosphonate therapy are lacking, future studies could explore whether targeting RANK/RANKL could ameliorate aneurysm progression driven by some forms of clonal hematopoiesis.

Collectively we show that clonal hematopoiesis was prevalent among patients with aortic aneurysm and that carriers experienced significantly more rapid aneurysm expansion. Peripheral blood clonal hematopoiesis screening could therefore serve as a minimally invasive biomarker, identifying high-risk individuals who merit closer imaging surveillance of aortic dilation. Beyond risk stratification, the mechanistic link between clonal hematopoiesis and aortic aneurysm shown here suggests tractable therapeutic avenues. We found that the bisphosphonate alendronate and anti-RANKL antibody, which are typically used to treat bone diseases by inhibiting osteoclast differentiation and activity, were effective in attenuating aortic aneurysm progression in the animal model. Because this anti-resorptive agent is approved for osteoporosis, the repurposing of these drugs for treating aortic dilation in patients positive for clonal hematopoiesis could enable mutation-guided therapy for this otherwise intractable disease. Intriguingly, Dnmt3a-mediated clonal hematopoiesis has also been shown to exacerbate osteoporosis by activating osteoclasts (18), which aligns with our current findings.

### Study limitations.

Although our study provides important insights into the role of Tet2-driven clonal hematopoiesis in aortic aneurysms, several limitations should be acknowledged. First, while we documented the role of macrophages, we cannot rule out the potential contribution of other immune cell subsets. Second, although we delineated a RANK/RANKL-dependent, osteoclast-like program as a key pathogenic axis, *Tet2*-mutant macrophages also released proinflammatory cytokines that may have acted in parallel or synergistically. This possibility can be addressed by future analyses. Third, the human study relied on a relatively small, single-ethnicity cohort and an observational design, limiting statistical power. Nonetheless, our use of high-fidelity, error-corrected NGS enabled more sensitive detection of clonal hematopoiesis mutations than conventional biobank studies, and murine loss-of-function experiments provide causal evidence of the pathogenic potential of clonal hematopoiesis. Fourth, longitudinal imaging data on aneurysm growth were available only for a subset of patients, but this subgroup appeared broadly comparable to the overall cohort. Fifth, our cohort was limited to patients who underwent consecutive endovascular aneurysm repair (EVAR); therefore, the generalizability of our findings to patients undergoing open repair requires further study. Sixth, because clonal hematopoiesis was assessed at the time of surgery and longitudinal clonal hematopoiesis measurements were not available, we cannot fully exclude reverse causality. Finally, clonal hematopoiesis as a whole, but not individual mutations, was associated with accelerated aneurysm progression, likely reflecting the relatively small sample size and genetic heterogeneity of individual driver mutations. However, mechanistic data from the Tet2 mouse model of clonal hematopoiesis indicate that this driver gene is causal for accelerated aneurysm expansion.

### Conclusions.

In summary, our data, to our knowledge, identify clonal hematopoiesis as a previously unrecognized contributor to aortic aneurysm progression. Our mechanistic studies involving the *Tet2* gene revealed that clonal hematopoiesis promoted AAA progression via macrophage reprogramming toward an osteoclast-like, matrix-degrading phenotype. Targeting the RANK/RANKL axis, therefore, represents a tractable precision-medicine strategy for this otherwise intractable disease.

## Methods

### Sex as a biological variable.

This study examined male mice because male animals exhibited less variability in phenotype. It is unknown whether the findings are relevant for female mice.

This study included both male and female participants. The sex distribution is presented in Table 1. No sex-specific analyses were conducted, and the findings are considered to be broadly applicable across sexes.

### Clinical data.

This study was a retrospective observational cohort study. All participant data (including standard baseline demographics, laboratory data, and medical histories) were sourced from a Nagoya University cohort in Nagoya, Japan, and patients were included consecutively between December 2023 and February 2025. Race and ethnicity were self-reported, and all participants were Japanese. Blood samples for ultra-deep, error-corrected sequencing were collected at the time of surgical admission, and the aneurysm growth rate prior to surgery was analyzed retrospectively. All patients were monitored with contrast-enhanced CT every 6–12 months according to standard clinical practice. All 44 patients included in this study had AAAs; thoracic aneurysms were not included. Longitudinal imaging data for aneurysm diameter were available for 21 of 44 patients. Patients with any clinical features suggestive of hereditary or syndromic aortopathies were excluded. Measurement of the aortic diameter in the patient cohort was done using contrast-enhanced CT, the standard modality for preoperative evaluation of AAAs. The maximum aneurysm diameter was determined by a board-certified radiologist, who was blinded to clonal hematopoiesis status and clinical outcomes to ensure objective and unbiased assessment. Measurements were performed using axial and reconstructed sagittal images, and the largest cross-sectional diameter was recorded for analysis. This standardized approach was applied uniformly across all cases.

### Ultra-deep, error-corrected DNA sequencing.

Patient blood samples were collected at the time of surgical admission using PAXgene DNA tubes (catalog 761165, BD Biosciences) and cryopreserved until use. Ultra-deep, error-corrected DNA-seq was performed as previously described (20). Briefly, genomic DNA was isolated from the blood samples using QIAamp DNA extraction kits (catalog 51104; Qiagen). The pooled libraries were sequenced using a NovaSeq X instrument (Illumina). Raw data from biological replicate error-corrected sequencing were analyzed as previously described (20). Briefly, a minimum of 3 raw reads sharing the same unique molecular identifier were used to generate an error-corrected consensus sequence. The output was filtered to include bases with 700 times or greater consensus read coverage within the target regions of a custom gene panel (Supplemental Table 1), excluding common variants (minor allele fraction ≥0.01) identified by the 1000 Genomes Project (39). The selected gene panel incorporated well-documented clonal hematopoietic genes based on the IntoGen Clonal Hematopoiesis Mutation Browser database (40). For single-nucleotide variant calling, a position-specific binomial background error model was implemented for variant calling. Each genomic position was modeled independently by compiling the background error rate of normal samples for that specific genomic position, defined as the fraction of nonreference reads relative to the total number of sequencing reads at that position. For each individual sample, the number of reads supporting the nonreference allele at a given genomic position was compared with the corresponding background error rate. A genomic position was considered positive when the observed number of nonreference reads was significantly higher than the background error rate based on a binomial test (*P* < 0.01). Variants with a VAF of greater than 0.45 were removed to exclude potential germline variants, and those below this threshold occurring in 3 or more individuals were considered recurrent sequencing errors and omitted. Variants with a VAF of less than 0.005 were excluded because of decreased confidence in distinguishing variants due to intrinsic sequencing error. Finally, only frameshift, stop-gain, and missense variants were included in the downstream analysis, as these mutations have the potential to influence protein function.

### Mice.

B6(Cg)-*Tet2*^tm1.2Rao^/J (no. 023359), B6.129P2-*Apoe*^tm1Unc^/J (no. 002052), C57BL/6J WT (no. 000664), and B6(C)-Gt(ROSA)26Sorem1.1(CAG-cas9*,-EGFP)Rsky/J (no. 028555) mice were obtained from The Jackson Laboratory. B6.SJL-Ptprc^a^ Pepc^b^/BoyJ mice were obtained from Sankyo Labo Service Corporation.

### Cell culture.

For in vitro experiments, each biological replicate represents an independent culture derived from BM cells obtained from different mice. A Lenti-X 293T cell line was obtained from Takara Bio. Cells were cultured in DMEM supplemented with 10% FBS and penicillin/streptomycin/l-glutamine (complete medium) at 37°C in a humidified incubator with 5% CO_2_. BM-derived macrophages were isolated and cultured in complete medium (RPMI 1640 supplemented with 10% FBS; catalogs 11-875-093 and SH3091003, respectively; Thermo Fisher Scientific). First, the BM was flushed from the tibia and femurs of 8- to 10-week-old mice, and cells were washed and cultured overnight in complete medium. HSPCs were purified via differential plating and defined as the nonadherent population after 16 hours of culturing. Macrophage proliferation and differentiation were induced by culturing for 3 days in complete medium supplemented with macrophage CSF (M-CSF) (30 ng/mL; catalog 315-02, PeproTech). Cells were then detached using 0.25% trypsin with EDTA (catalog 25200056, Thermo Fisher Scientific) at 37°C for 30 minutes to 1 hour and reseeded in new plates containing M-CSF and RANKL (25 or 50 ng/mL; catalog 315-11C, PeproTech). The cells were further incubated for 3 days in complete medium. To inhibit macrophage differentiation, alendronate sodium (0.5 μg/mL; catalog A4978, MilliporeSigma) was added at the time of reseeding. Differentiation into osteoclast-like cells was assessed using a TRAP staining kit (catalog AK04F, Cosmo Bio).

### Lentivirus production.

The plasmids pLKO5.sgRNA.EFS.tRFP (catalog 57823), psPAX2 (catalog 12260), and pMD2.G (catalog 12259) were purchased from Addgene. A sgRNA targeting exon 9 of the *Rank* gene was designed using the CRISPR design tool (https://www.idtdna.com/site/order/designtool/index/CRISPR_CUSTOM). A sgRNA targeting mouse *Rank* (AGATTCTAGGACGTTCACAC) or a noncoding sgRNA targeting an intron in the murine *Actb* gene (AGGTTGCTCTGACAACCACA) were subcloned into the BsmB1 restriction enzyme site of the appropriate vector. Lentivirus particles were generated as previously described (41). Briefly, the plasmids (lentiviral vector, psPAX2, and pMD2.G) were cotransfected into Lenti-X 293T cells using polyethyleneimine. The culture supernatant was collected 48 hours after transfection, filtered through a 0.45 μm filter, and concentrated by ultracentrifugation at 72 ,100*g* for 3 hours. The virus pellet was resuspended in StemSpan medium (catalog 09600, STEMCELL Technologies) and stored at –80°C. Lentiviral titers were determined using a Lenti-X quantitative reverse transcription PCR (qRT-PCR) Titration Kit (catalog 631235, Clontech).

### Isolation of lineage-negative BM cells and lentivirus transduction.

A lineage depletion kit (catalog 130-090-858, Miltenyi Biotech) was used to isolate lineage-negative BM cells from mice that had been generated by crossing B6(C)-Gt(ROSA)26Sorem1.1(CAG-cas9*,-EGFP)Rsky/J and B6(Cg)-*Tet2*^tm1.2Rao^/J mice, and selecting for heterozygous alleles of both strains. Cells were preincubated with StemSpan medium (catalog 09600, STEMCELL Technologies) for 1.5 hours at 37°C. Lentivirus transduction was performed in 20 ng/mL thrombopoietin (catalog 315-14, PeproTech), 50 ng/mL stem cell factor 1 (SCF-1) (catalog 250-03, PeproTech), and 4 μg/mL polybrene (catalog 12996-81, Nacalai Tesque) for 16–20 hours. The cells were collected and resuspended in RPMI medium before transplantation.

### BMT.

Recipient mice were exposed to 2 radiation doses of 4.5 Gy at 4-hour intervals using MBR-1618R-BE (Hitachi). For mouse models of clonal hematopoiesis, BM cells containing 20% *Tet2*-KO and 80% WT cells (5 × 10^6^ cells in 200 μL RPMI 1640 medium/mouse) were retro-orbitally injected into 8- to 10-week-old *Apoe*-KO mice. For RNA-seq experiments, 100% *Tet2*-KO or 100% WT donor BM was used. To distinguish between donor *Tet2*-KO and WT cells, WT cells were obtained from mice carrying the CD45.1 variant of the CD45 hematopoietic antigen, whereas *Tet2*-KO cells were obtained from mice carrying the CD45.2 variant. Control mice (20% WT BMT) were transplanted with 20% CD45.2^+^ and 80% CD45.1^+^ WT cells. For lineage-negative cell transplantation, lentivirus-transduced cells (5 × 10^5^ cells in 200 μL RPMI 1640 medium/mouse) were used instead. The experimental mice were randomly assigned to either the experimental or control group. We did not exclude any mice with the exception of those that were not used due to human error. Although no power analyses were performed to determine sample sizes, appropriate sample sizes for statistical analysis were selected on the basis of our previous experimental findings using the same models.

### qRT-PCR.

Total RNA from tissues and cultured cells was isolated using QIAzol reagent (catalog 79306, Qiagen) and a NucleoSpin RNA Plus kit (catalog 740984.50, Takara). RNA (0.5–1.2 μg) was reverse transcribed with a QuantiTect Reverse Transcription Kit (catalog 205313, Qiagen). qRT-PCR was performed with Power SYBR Green reagent (catalog 4368708, Thermo Fisher Scientific) on a ViiA7 PCR system. A standard thermocycling protocol (95°C for 15 seconds and 60°C for 60 seconds, 40 cycles total) was used for gene amplification. Primer sequences included 5′-GCTCCAAGCAGATGCAGCA-3′ and 5′-CCGGATGTGAGGCAGCAG-3′ (*36b4*), 5′-GCGACCATTGTTAGCCACATACG-3′ and 5′-CGTTGATGTCGCACAGAGGGAT-3′ (*Trap*), and 5′-CTGGACAGCCAGACACTAAAG-3′ and 5′-CTCGCGGCAAGTCTTCAGAG-3′ (*Mmp9*). Gene expression was analyzed using the ΔΔCt method and normalized according to that of the reference gene *36b4*.

### Hematopoietic cell and flow cytometric analyses of peripheral blood and aortic immune cells.

Hematopoietic parameters were analyzed using a VETSCAN HM5 (Zoetis). Flow cytometric analysis of peripheral blood leukocytes and aortic immune cells was performed at the indicated time points as previously described (41). Peripheral blood cells were obtained from the retro-orbital vein and collected in K2 EDTA–containing Fuji tubes (catalog A01032, Fujifilm). RBCs were lysed in eBioscience 1X RBC Lysis Buffer (catalog 00-4333-57, Thermo Fisher Scientific) for 5 minutes on ice. Incubation with antibodies was performed for 20 minutes at room temperature in the dark. Aortic tissues were minced and digested in collagenase I (450 U/mL), collagenase XI (125 U/mL), hyaluronidase (450 U/mL), and DNase I (60 U/mL) (catalog C0130, C7657, H3506, and D4513, respectively, MilliporeSigma) at 37°C for 30 minutes using a ThermoMixer C (Eppendorf) at 900 rpm. Aorta samples were subsequently homogenized using cell strainers (Falcon, catalog 352350, Thermo Fisher Scientific). After incubation with antibodies, dead cells were excluded from the analysis using Zombie Aqua or Violet (catalogs 423102 and 423113, respectively; BioLegend) according to the manufacturer’s instructions. To determine the cell numbers, 123count eBeads (catalog 01-1234-42, Thermo Fisher Scientific) were used. Data acquisition was performed using the Fortessa (BD Biosciences) and analyzed with FlowJo software (FlowJo), and cell numbers were normalized to the number of cells/100 mg wet weight of the aorta. Flow cytometric analysis was performed using distinct antibody panels according to sample type and immune cell subsets, as detailed in Supplemental Table 2. The cells were defined as described in the gating strategy shown in Supplemental Figure 2.

### Echocardiographic analyses.

Echocardiography was performed on isoflurane-anesthetized mice. Mice were kept semi-awake in a shallow state of anesthesia by monitoring their responses to physical stimuli (tail pinch, etc.), and heart rate was maintained at 500–600 bpm. M-mode images of the abdominal aorta in the supraceliac region just above the bifurcation of the celiac artery were obtained using a Vevo 3100 imaging system (FUJIFILM VisualSonics) equipped with an MS400 (18-38 MHz) phased-array transducer.

The aortic diameter was assessed using high-resolution M-mode echocardiography at a standardized anatomical location immediately distal to the celiac arteries (42). The celiac artery was first identified in the long-axis view and centered on the screen, after which the probe was rotated 90° to obtain a consistent short-axis view. M-mode tracings were acquired at the site demonstrating the maximal vessel diameter. Measurements were performed at end-systole across 3 consecutive cardiac cycles. The aortic diameter was defined as the distance from the midpoint of 1 medial layer to the midpoint of the opposite medial layer, ensuring consistent depth and perpendicular orientation across animals.

### Pump implantation.

To induce aortic aneurysms in mice, osmotic minipumps (Alzet model 2004) containing either AngII (1.44 mg/kg/d, diluted in sterile saline, catalog A9525, MilliporeSigma) or saline (sham) were implanted s.c. into a small pocket made through an incision at the nape of the neck. The mice were anesthetized with isoflurane during the entire surgical procedure, and the wounds were closed with wound clips. Osmotic minipumps were primed in PBS at 37°C for 24 hours before implantation, and they remained in place for 28 days after implantation. In some experiments, the mice were implanted with a second minipump to prolong the time course. Blood pressure was measured using tail-cuff plethysmography, as previously described (41).

### Alendronate treatment.

For the bisphosphonate experiment, mice were s.c. injected twice a week with alendronate sodium (catalog A4978, MilliporeSigma) at 100 μg/kg. Oral alendronate (1.4 mg/kg daily) was administered by gavage after a 4-hour fast, with food withheld for 30 minutes after dosing, beginning on day 0 of the AngII infusion.

### RANKL-blocking interventions in mice.

Anti-RANKL monoclonal antibody (InVivoMAb anti-mouse RANKL, clone IK22/5; BE0191) was administered i.p. at 4 mg/kg per injection, once every 2 days. A rat IgG2a isotype control antibody (InVivoMAb, clone 2A3; BE0089) was used as the control.

### Histology.

Aortic tissues were perfused with cold PBS from the transected end and fixed in 10% formalin at 4°C overnight. Samples were processed for paraffin embedment and cut into 4 μm thick sections. For tissue staining, the sections were deparaffinized and rehydrated. To determine elastic fibers, aortic sections were stained with a Microscopy Elastica van Gieson staining kit (catalog 1.15974.0002, MilliporeSigma). The images acquired using a BZ-X710 Keyence microscope were analyzed with ImageJ software (NIH) to quantify elastic fiber thickness and the number of tears. Elastin fiber thickness and elastin rupture were quantified in EVG-stained abdominal aortic sections. One section per mouse, corresponding to the maximally dilated aneurysmal segment, was analyzed. Five nonoverlapping high-power fields were selected per section in a blinded manner. Elastin thickness was measured at 3 predefined points per field along the medial elastic lamellae and averaged to obtain a single value for each mouse. Elastin rupture was semiquantitatively scored according to the percentage of disrupted elastic lamellae in each field, and the mean score across 5 fields was used for analysis. All measurements were performed by 2 independent investigators blinded to genotype and treatment group. For TRAP^+^ cell quantification, 5 nonoverlapping high-power fields per well were analyzed, and TRAP^+^ cells were manually counted by 2 independent observers blinded to the genotype and treatment group. Counts were averaged across fields to obtain a single value per well.

### Western blotting.

BM-derived macrophages were lysed directly in culture dishes using SDS sample buffer (Blue Loading Buffer, catalog B7703S, New England BioLabs) containing 40 mM DTT, followed by incubation at 95°C for 5 minutes. Cell lysates were separated using SDS-PAGE (catalog 4561033, Bio-Rad Laboratories) and transferred onto a PVDF membrane. After blocking with 5% skim milk in PBS with Tween 20 (0.1%) for 1 hour, the membranes were incubated with the indicated antibodies overnight at 4°C, followed by an HRP-conjugated second antibody (catalog sc-2357, Santa Cruz Biotechnology) for 1 hour at room temperature. The following antibodies were used for immunoblotting: anti–β-actin (13E5) rabbit monoclonal antibody (catalog 4970, Cell Signaling Technology) and anti-RANK (EPR26196-15) rabbit monoclonal antibody (catalog ab305233, Abcam). Images were visualized using an ECL Prime Western Blotting System (catalog RPN2232, GE Healthcare).

### Immunostaining.

For smooth muscle actin, CD68, and PECAM-1 staining, aortic tissue sections were deparaffinized, and antigen retrieval was performed using Epitope Retrieval Solution, pH 6 (catalog RE7113-CE, Leica Biosystems). Sections were blocked with Protein Block Serum-Free (catalog X0909, Agilent Technologies) for 30 minutes and subsequently incubated with a primary antibody overnight at 4°C. To distinguish target staining from the background, a secondary antibody was used as a negative control in each experiment. After washing with Tris-buffered saline, sections were incubated with secondary antibodies for 2 hours at room temperature. Nuclei were stained with DAPI. Fluorescence images were captured using a BZ-X710 Keyence microscope. The primary antibodies included mouse anti–actin α-smooth muscle-Cy3 (1A4) (catalog C6198, MilliporeSigma), anti–rabbit CD68 (E307V) (catalog 97778, Cell Signaling Technology), and anti–goat CD31/PECAM-1 (catalog AF3628, R&D Systems). The secondary antibodies included donkey anti–rabbit Alexa Fluor 488 (catalog A21206, Thermo Fisher Scientific) and donkey anti–goat Alex Fluor 647 (catalog A21447, Thermo Fisher Scientific).

### Cell sorting.

For qRT-PCR analysis of aortic macrophages (CD45^+^Ly6G^–^CD64^+^Ly6C^–^), aorta digests were prepared as described for the flow cytometric analysis, and sorting was performed on a FACSAria Fusion cell sorter (BD Biosciences) with a 100 μM nozzle and flow pressure set to 20 psi. A total of 20,000 cells were sorted for each population.

### RNA-seq.

Total RNA was extracted from the FACS-sorted aortic macrophages using an RNeasy Micro Kit (Qiagen). RNA integrity and concentration were assessed using a bioanalyzer (Agilent Technologies). RNA-seq library preparation was outsourced to Takara Bio Inc. Libraries were prepared using a SMART-Seq v4 Ultra Low Input RNA Kit combined with a Nextera XT DNA Library Prep Kit optimized for low-input polyA+ RNA. Sequencing was performed using an Illumina NovaSeq platform, which generated approximately 40 million paired-end reads per sample. The mouse GRCm39 (mm39) construct was used as the reference genome for alignment. Sequencing data, including read alignment, quantification, and expression of known transcripts, were processed using Expression Miner 2.0 (Takara Bio). For further downstream analysis, read count data were imported into R (version 4.2.2), and differential expression analysis was conducted using the DESeq2 package. Genes with an adjusted value of less than 0.05 and |log_2_ fold change| of greater than 1 were considered differentially expressed. Functional enrichment analyses (Gene Ontology [GO] and Kyoto Encyclopedia of Genes and Genomes [KEGG]) were performed using clusterProfiler, and gene set enrichment analysis (GSEA) was carried out using the fgsea and MSigDB hallmark gene sets.

### Statistics.

For experimental (in vivo and in vitro) studies, all statistical analyses were performed using GraphPad Prism 10 (GraphPad Software). Data are presented as the mean ± SEM. Data distribution was assessed using the Shapiro-Wilk normality test. For normally distributed data with 1 experimental variable, statistical analyses were performed using parametric tests: unpaired (2-tailed) Student’s *t* test for 2 groups with equal variance, or Welch’s *t* test for 2 groups with unequal variance, and 1-way ANOVA with Tukey’s multiple-comparison test for more than 2 groups. Data with 2 independent variables were evaluated using 2-way ANOVA or 2-way, repeated-measures ANOVA, as appropriate, followed by Šidák’s multiple-comparison post hoc tests. For non-normally distributed data with 1 experimental variable, statistical analyses were performed using nonparametric tests: the Mann-Whitney *U* (2-tailed) test for 2 groups and Kruskal-Wallis with Dunn multiple-comparison post hoc tests for more than 2 groups. Statistical significance was set at a *P* value of less than 0.05. Statistical analyses were performed using biological replicates as independent samples. Statistical significance is shown only for selected pairwise comparisons relevant to the hypothesis.

For analyses of human clinical samples, continuous variables are summarized as the mean ± SD, and categorical variables as a number (percentage). Between-groups differences were assessed using 2-tailed Student’s *t* test or the Mann-Whitney *U* test for continuous variables and Pearson’s χ^2^ test or Fisher’s exact test for categorical variables, as appropriate. The association between clonal hematopoiesis status and aneurysm growth rate was evaluated using univariable linear regression. All *P* values are 2 sided, and a *P* value of less than 0.05 was considered statistically significant. Statistical analyses were performed using GraphPad Prism 10.

### Study approval.

The study protocols were approved by the IACUC of Nagoya University (approval no. M250302-002). The study investigating the association between clonal hematopoiesis and AAA was registered with the University hospital Medical Information Network (UMIN) Clinical Trials Registry (UMIN000052118). Written informed consent was obtained from all participants prior to inclusion in the study.

### Data availability.

Supporting data values for all graphs and values underlying the reported means are provided in the Supporting Data Values file. All raw and processed RNA-seq data generated in this study have been deposited in the DDBJ Sequence Read Archive (DRA) under accession number DRP016820 (BioProject: PRJDB39638). The full RNA-seq dataset used for differential expression analysis and volcano plot generation is provided in Supplemental Data 1. Clinical genomic data derived from human samples have been deposited in the NBDC Human Database under controlled access (accession number, study: JGAS000864; dataset: JGAD001007). Access to these data requires approval from the NBDC Data Access Committee in accordance with institutional and national guidelines.

## Author contributions

JY and YY conducted the majority of the experiments, acquired and analyzed the data, and wrote the manuscript. JL, KK, TH, RO, MK, EMY, KH, KDM, and TE contributed to data acquisition and interpretation of experimental results. YK, SI, and HB collected patient data. KW and MT provided conceptual advice and overall study guidance. KW also provided the mice used in this study and revised the manuscript. YY, MT, and TM provided research funding. TM supervised the project. All authors reviewed and approved the final manuscript.

## Conflict of interest

The authors have declared that no conflict of interest exists.

## Funding support

- Japan Society for the Promotion of Science KAKENHI (grants 22K16136 and 24K19026).
- Japan Science and Technology Agency (JST) Fusion Oriented Research for Disruptive Science and Technology (FOREST) Program (grant JPMJFR2217).
- Japan Agency for Medical Research and Development (AMED) (grant JP256f0137010)
- Japan Foundation for Applied Enzymology.
- Kowa Life Science Foundation.
- MSD Life Science Foundation.
- Mitsubishi Foundation.
- Suzuken Memorial Foundation.
- The Hori Science and Arts Foundation.
- Mochida Foundation.
- Takeda Science Foundation.
- Sakakibara Heart Foundation.
- Japan Heart Foundation.
- Nippon Shinyaku Co., Ltd.
- Senshin Medical Research Foundation (to YY).
- Japan Society for the Promotion of Science KAKENHI (grants 23K27594, to MT and 24K02444, to TM).

## Supplementary Material

Supplemental data

Supplemental data set 1

Unedited blot and gel images

Supporting data values

## Figures and Tables

**Table 2:**
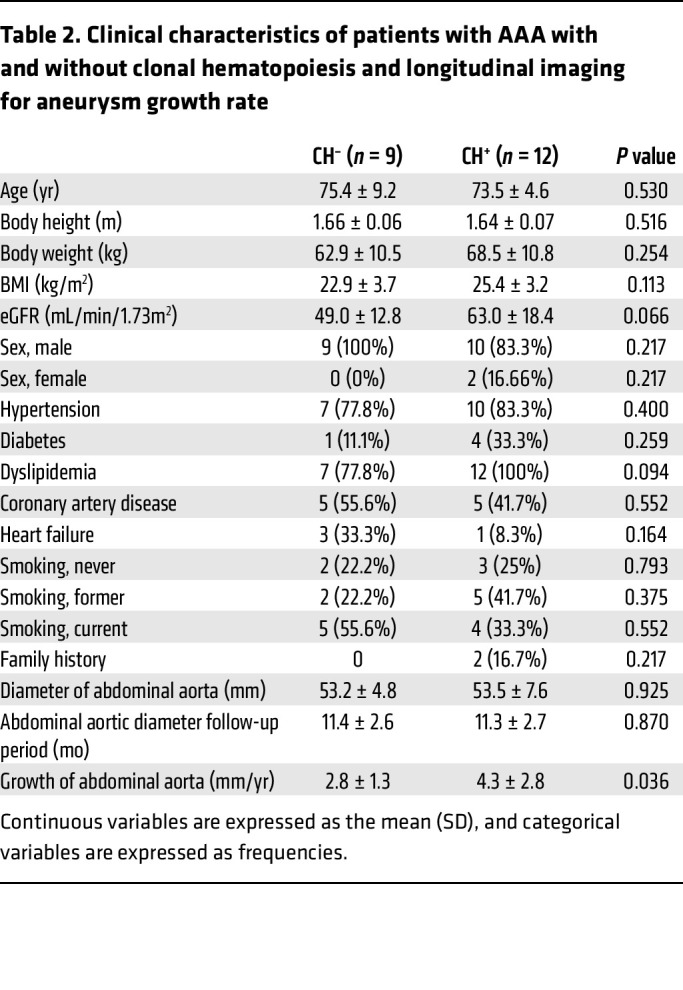
Clinical characteristics of patients with AAA with and without clonal hematopoiesis and longitudinal imaging for aneurysm growth rate

**Table 1:**
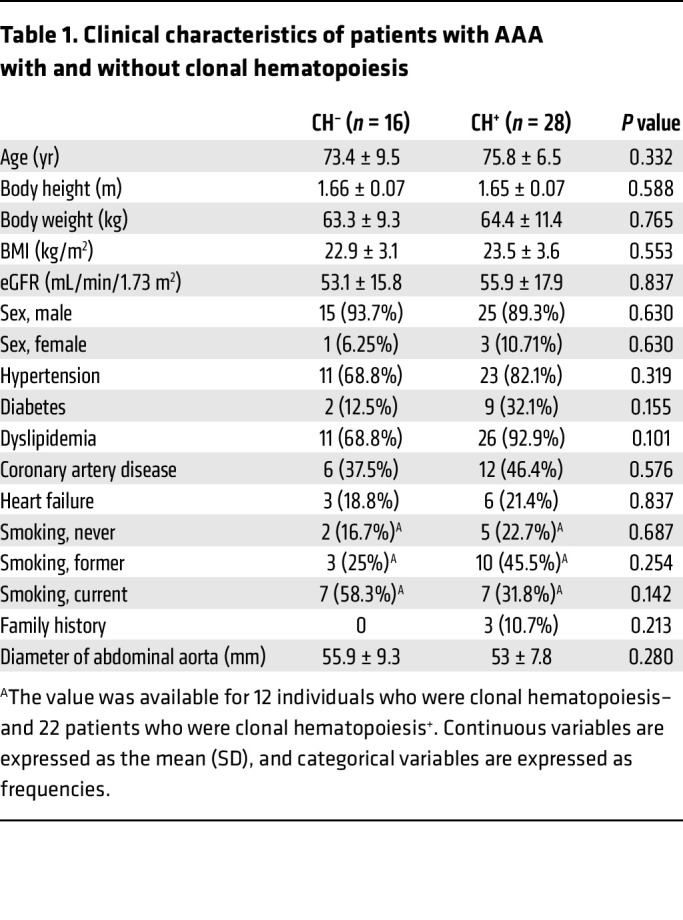
Clinical characteristics of patients with AAA with and without clonal hematopoiesis

## Version 1 02/25/2026

In-Press Preview

## Version 2 04/15/2026

Electronic publication

## References

[1] Kent KC (2014). Clinical practice. Abdominal aortic aneurysms. N Engl J Med.

[2] Golledge J (2020). Lack of an effective drug therapy for abdominal aortic aneurysm. J Intern Med.

[3] Nordon IM (2011). Pathophysiology and epidemiology of abdominal aortic aneurysms. Nat Rev Cardiol.

[4] King SJ (2025). Heart disease mortality in the United States, 1970 to 2022. J Am Heart Assoc.

[5] Golledge J (2023). Pathogenesis and management of abdominal aortic aneurysm. Eur Heart J.

[6] Wanhainen A (2016). Surrogate markers of abdominal aortic aneurysm progression. Arterioscler Thromb Vasc Biol.

[7] Quintana RA, Taylor WR (2019). Cellular mechanisms of aortic aneurysm formation. Circ Res.

[8] Dale MA (2015). Inflammatory cell phenotypes in AAAs: their role and potential as targets for therapy. Arterioscler Thromb Vasc Biol.

[9] Shimizu K (2006). Inflammation and cellular immune responses in abdominal aortic aneurysms. Arterioscler Thromb Vasc Biol.

[10] Ladich E (2016). Vascular diseases: aortitis, aortic aneurysms, and vascular calcification. Cardiovasc Pathol.

[11] Evans MA, Walsh K (2023). Clonal hematopoiesis, somatic mosaicism, and age-associated disease. Physiol Rev.

[12] Walsh K (2024). The emergence of clonal hematopoiesis as a disease determinant. J Clin Invest.

[13] Steensma DP (2015). Clonal hematopoiesis of indeterminate potential and its distinction from myelodysplastic syndromes. Blood.

[14] Fuster JJ, Walsh K (2018). Somatic mutations and clonal hematopoiesis: unexpected potential new drivers of age-related cardiovascular disease. Circ Res.

[15] Yura Y (2020). Clonal hematopoiesis: a new step linking inflammation to heart failure. JACC Basic Transl Sci.

[16] Wong WJ (2023). Clonal haematopoiesis and risk of chronic liver disease. Nature.

[17] Miller PG (2022). Association of clonal hematopoiesis with chronic obstructive pulmonary disease. Blood.

[18] Kim PG (2021). Dnmt3a-mutated clonal hematopoiesis promotes osteoporosis. J Exp Med.

[19] Daugherty A (2000). Angiotensin II promotes atherosclerotic lesions and aneurysms in apolipoprotein E-deficient mice. J Clin Invest.

[20] Cochran JD (2023). Clonal hematopoiesis in clinical and experimental heart failure with preserved ejection fraction. Circulation.

[21] Jaiswal S (2014). Age-related clonal hematopoiesis associated with adverse outcomes. N Engl J Med.

[22] Kelly MJ (2019). Osteoclast-like cells in aneurysmal disease exhibit an enhanced proteolytic phenotype. Int J Mol Sci.

[23] Liu C (2010). Structural and functional insights of RANKL-RANK interaction and signaling. J Immunol.

[24] Nakagawa N (1998). RANK is the essential signaling receptor for osteoclast differentiation factor in osteoclastogenesis. Biochem Biophys Res Commun.

[25] Yamamoto M (2021). TNF receptor-associated factor 6 (TRAF6) plays crucial roles in multiple biological systems through polyubiquitination-mediated NF-κB activation. Proc Jpn Acad Ser B Phys Biol Sci.

[26] Luckman SP (1998). Nitrogen-containing bisphosphonates inhibit the mevalonate pathway and prevent post-translational prenylation of GTP-binding proteins, including Ras. J Bone Miner Res.

[27] Lacey DL (1998). Osteoprotegerin ligand is a cytokine that regulates osteoclast differentiation and activation. Cell.

[28] Wang W (2020). The interaction between lymphoid tissue inducer-like cells and T cells in the mesenteric lymph node restrains intestinal humoral immunity. Cell Rep.

[29] Fuster JJ (2017). Clonal hematopoiesis associated with TET2 deficiency accelerates atherosclerosis development in mice. Science.

[30] Sano S (2018). Tet2-mediated clonal hematopoiesis accelerates heart failure through a mechanism involving the IL-1β/NLRP3 inflammasome. J Am Coll Cardiol.

[31] Polizio AH (2024). Experimental TET2 clonal hematopoiesis predisposes to renal hypertension through an inflammasome-mediated mechanism. Circ Res.

[32] Zhang Q (2015). Tet2 is required to resolve inflammation by recruiting Hdac2 to specifically repress IL-6. Nature.

[33] Cull AH (2017). Tet2 restrains inflammatory gene expression in macrophages. Exp Hematol.

[34] Duplomb L (2008). Interleukin-6 inhibits receptor activator of nuclear factor kappaB ligand-induced osteoclastogenesis by diverting cells into the macrophage lineage: key role of Serine727 phosphorylation of signal transducer and activator of transcription 3. Endocrinology.

[35] Zhou P (2022). Cytokine-mediated immunomodulation of osteoclastogenesis. Bone.

[36] Longo GM (2002). Matrix metalloproteinases 2 and 9 work in concert to produce aortic aneurysms. J Clin Invest.

[37] Thompson RW (1995). Production and localization of 92-kilodalton gelatinase in abdominal aortic aneurysms. An elastolytic metalloproteinase expressed by aneurysm-infiltrating macrophages. J Clin Invest.

[38] Jones GT (2017). Meta-analysis of genome-wide association studies for abdominal aortic aneurysm identifies four new disease-specific risk loci. Circ Res.

[39] Auton A (2015). A global reference for human genetic variation. Nature.

[40] Pich O (2022). Discovering the drivers of clonal hematopoiesis. Nat Commun.

[41] Yura Y (2021). The cancer therapy-related clonal hematopoiesis driver gene Ppm1d promotes inflammation and non-ischemic heart failure in mice. Circ Res.

[42] Trachet B (2015). Performance comparison of ultrasound-based methods to assess aortic diameter and stiffness in normal and aneurysmal mice. PLoS One.

