# Supplementary material for: Tet2-driven clonal hematopoiesis drives aortic aneurysm via macrophage-to-osteoclast–like differentiation
Source: J Clin Invest. 2026 Feb 25;136(8):e198708. doi: 10.1172/JCI198708 (PMC13078872; doi:10.1172/JCI198708)
Supplement: Supplemental data [file jci-136-198708-s021.pdf]

Fig.S1

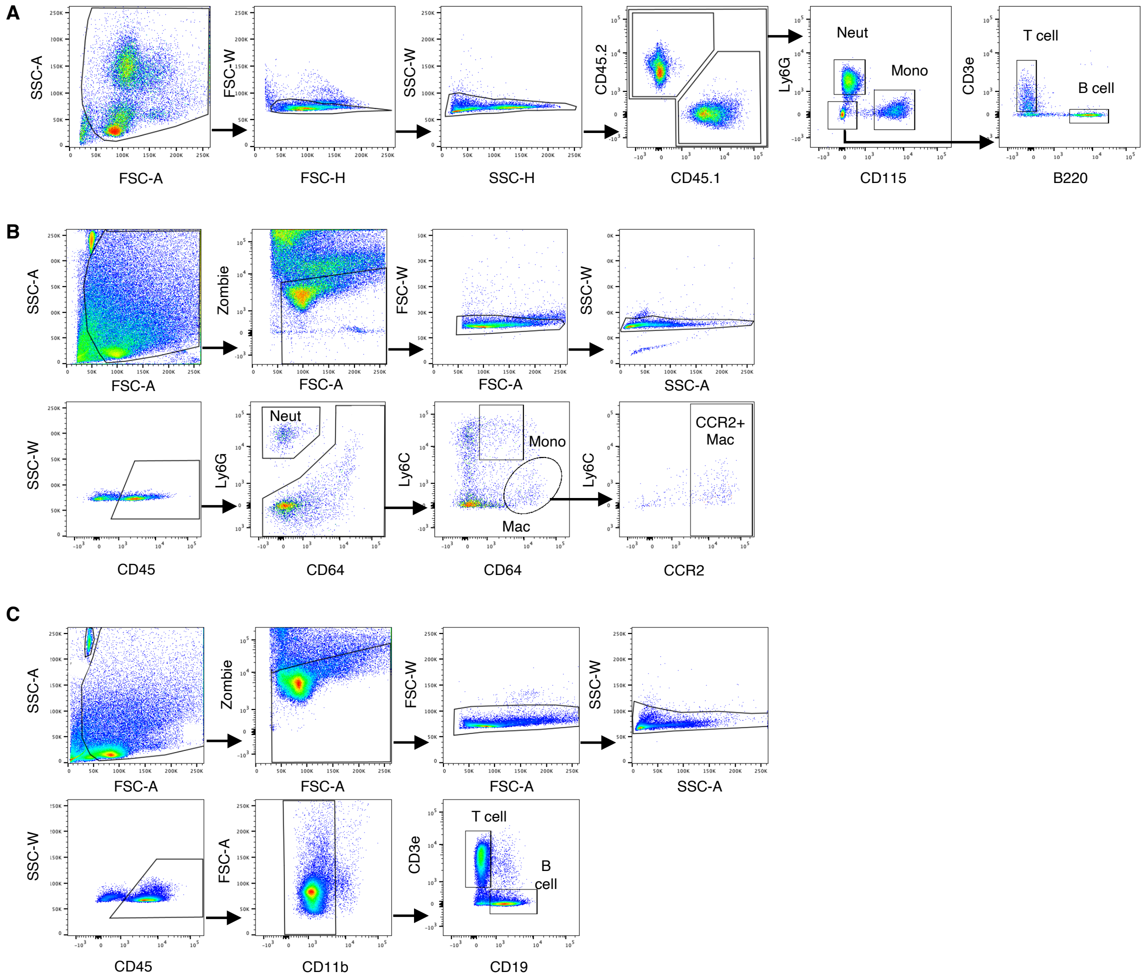

Fig. S1 Gating strategy for flow cytometry analysis in CD45.1/CD45.2 bone marrow chimeric mice. (A) Flow cytometry gating strategy for analysis of peripheral blood cells. Monocytes were defined as $CD45^{+}CD115^{+}$ cells, neutrophils as $CD45^{+}Ly6G^{+}$ cells, B cells as $CD45^{+}CD115^{-}Ly6G^{-}B220^{+}$ cells, and T cells as $CD45^{+}CD115^{-}Ly6G^{-}CD3e^{+}$ cells. **(B)** Flow cytometry gating strategy for analysis of myeloid cells in the aortic tissue. Monocytes were defined as $CD45^{+}Ly6G^{-}CD64^{int}Ly6C^{high}$ cells, neutrophils as $CD45^{+}CD64^{-}Ly6G^{+}$ cells, and macrophages as $CD45^{+}Ly6G^{-}CD64^{+}Ly6C^{low}$ cells. $CCR2^{-}$ macrophages were defined as $CD45^{+}Ly6G^{-}CD64^{+}Ly6C^{low}CCR2^{-}$ cells, and $CCR2^{+}$ macrophages as $CD45^{+}Ly6G^{-}CD64^{+}Ly6C^{low}CCR2^{+}$ cells. **(C)** Flow cytometry gating strategy for analysis of lymphoid cells

in the aortic tissue. T cells were defined as $CD45^{+}CD11b^{-}CD19^{-}CD3e^{+}$ cells, and B cells as $CD45^{+}CD11b^{-}CD19^{+}CD3e^{-}$ cells.

Fig.S2

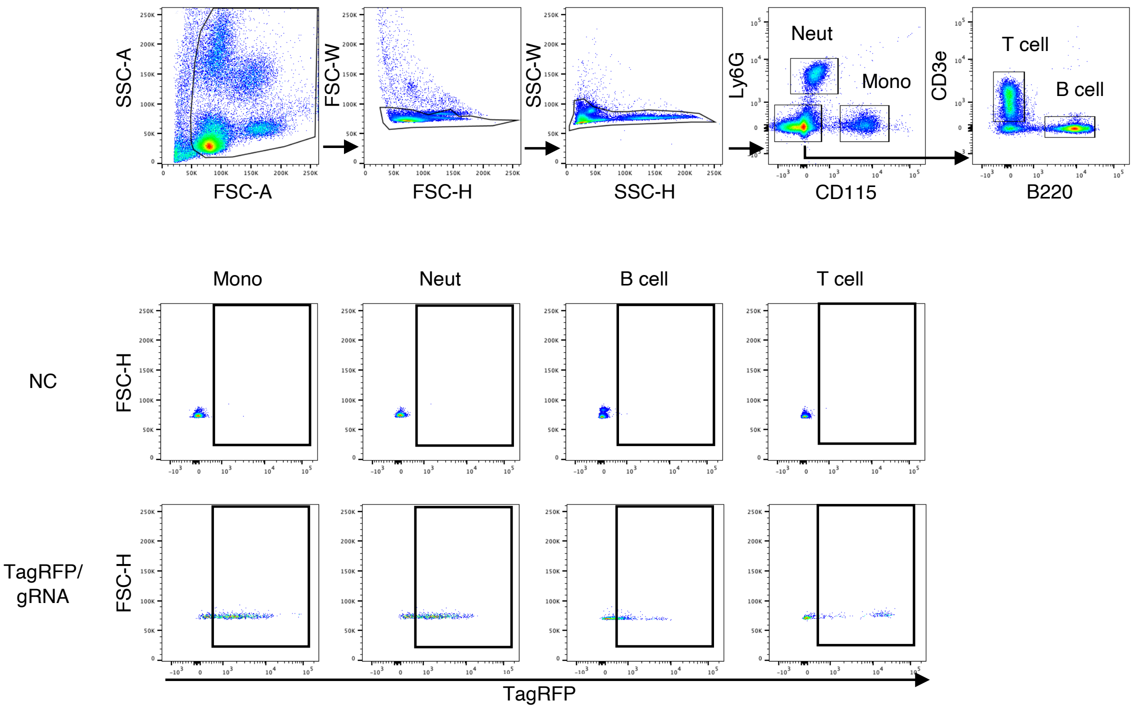

Fig. S2 Gating strategy for flow cytometry analysis in mice reconstituted with lentivirus-transduced hematopoietic stem cells. Flow cytometry gating strategy for analysis of peripheral blood cells. Monocytes were defined as $CD115^+$ cells, neutrophils as $Ly6G^+$ cells, B cells as $CD115^-Ly6G^-B220^+$ cells, and T cells as $CD115^-Ly6G^-CD3e^+$ cells. Lentivirus-transduced cells expressing sgRNA were identified as TagRFP-positive. NC indicates the negative control.

Fig.S3

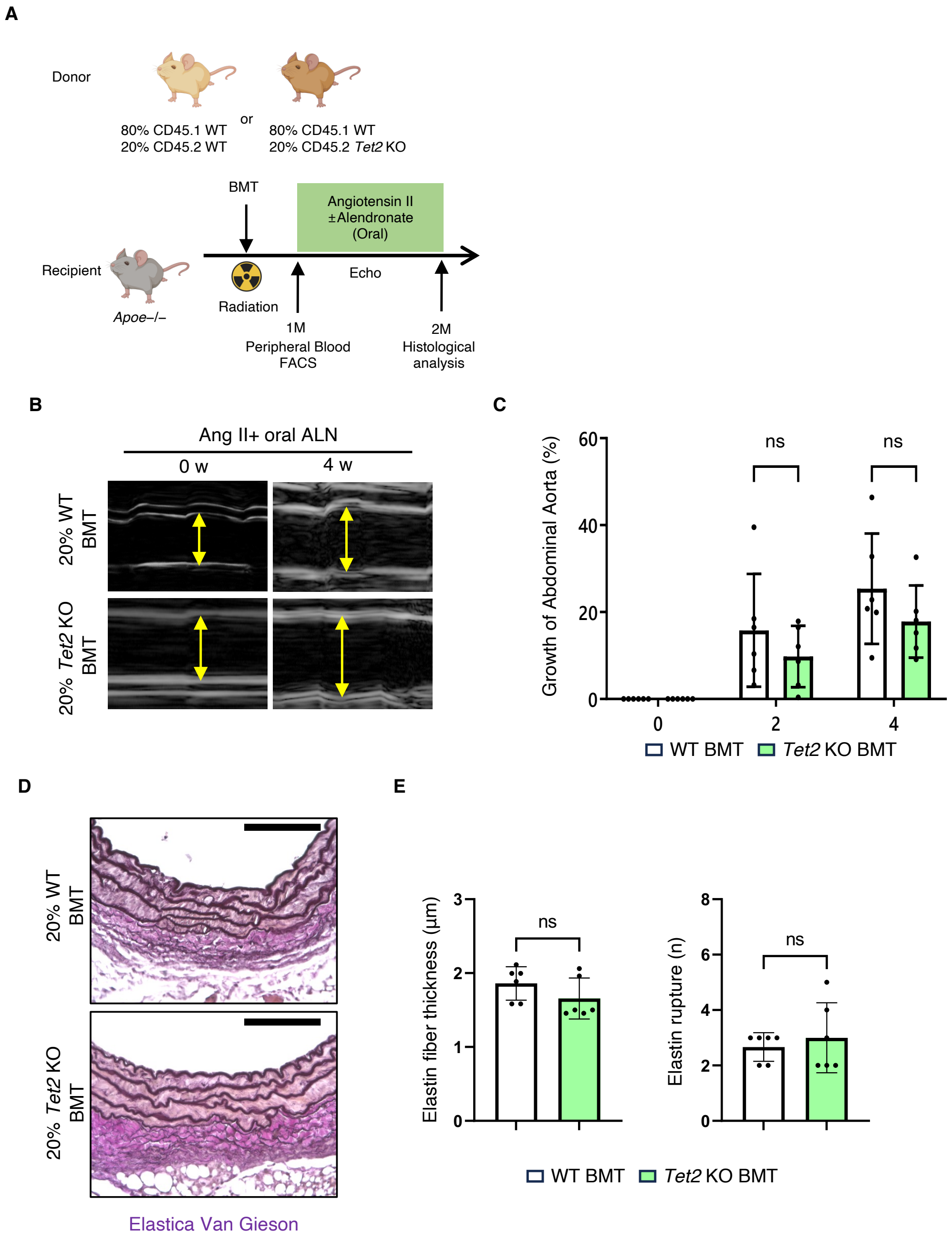

Fig. S3 Pharmacological inhibition of tartrate-resistant acid phosphatase type 5 (TRAP)-positive macrophage by oral alendronate (ALN) ameliorates abdominal aortic aneurysm (AAA) phenotype in mice with clonal hematopoiesis. (A) Schematic of in vivo experimental design using clonal hematopoiesis and abdominal aortic aneurysm model with oral ALN treatment. **(B)** Representative

ultrasound images of the abdominal aorta at 0 (baseline) and 4 weeks post- angiotensin II (AngII) infusion (n = 6 mice per genotype). **(C)** Quantification of abdominal aortic diameter at 0 (baseline), 2, and 4 weeks post-AngII infusion with ALN treatment (n = 6 mice per genotype). Statistical significance was evaluated using two-way ANOVA with Sidak multiple comparison test. **(D, E)** Representative images of Elastica van Gieson staining of abdominal aortic tissue and quantification of elastin fiber thickness and elastin rupture (*n* = 6 mice per genotype) Scale bar = 100 μm. Statistical significance was evaluated using two-tailed unpaired Student's *t*-test. *P < 0.05; **P < 0.01; ***P < 0.001; ****P < 0.0001.

Table. S1

GENE	NCBI_REFERENCE_SEQUENCE	TARGET_EXONS
ASXL1	NM_015338	11-13
ATM	NM_000051	1-63
CBL	NM_005188	8,9
CEBPA	NM_004364	1
CHEK2	NM_001005735	2-15
DNMT3A	NM_022552	1-23
EZH2	NM_004456	2-20
GNB1	NM_002074	5-7
JAK2	NM_004972	12-16
PHF6	NM_001015877	2-10
PPM1D	NM_003620	6
RUNX1	NM_001122607	1,2,3,5,6,7,8,9
SF3B1	NM_012433	13-18
SMC1A	NM_006306	1-25
SRSF2	NM_003016	2
TET2	NM_001127208	3-11
TP53	NM_000546	1-11

Table. S1 Ultra-deep error-corrected sequencing gene panel

Table. S2

Peripheral blood 1

ANTIBODIES	FLUOROPHORE	CLONE	SOURSE	IDENTIFIER
Anti-CD45.1	PE-Cy7	A20	BioLegend	Cat# 110730
Anti-CD45.2	BV785	104	BioLegend	Cat# 109839
Anti-CD115	PE	AFS98	BioLegend	Cat# 135505
Anti-Ly6C	APC	HK1.4	BioLegend	Cat# 128015
Anti-Ly6G	PerCP-Cy5.5	1A8	BioLegend	Cat# 127616
Anti-B220	APC-Cy7	RA3-6B2	BioLegend	Cat# 103224
Anti-CD4	FITC	RM4-5	BioLegend	Cat# 100510
Anti-CD8a	BV510	SK1	BioLegend	Cat# 100751
Anti-CD3	PE/Dazzle594	UCHT1	BioLegend	Cat# 100347

Aorta Myeloid

ANTIBODIES	FLUOROPHORE	CLONE	SOURSE	IDENTIFIER
Anti-CD45.1	PE	A20	BioLegend	Cat# 110707
Anti-CD45.2	BV785	104	BioLegend	Cat# 109839
Anti-CD64	PE-Cy7	X54-5/7.1	BioLegend	Cat# 139313
Anti-CCR2	BV421	SA203G11	BioLegend	Cat# 150605
Anti-Ly6G	PerCP-Cy5.5	1A8	BioLegend	Cat# 127616
Anti-Ly6C	FITC	HK1.4	BioLegend	Cat# 128005
Live dead	Zombie aqua		BioLegend	Cat# 423102

Aorta Lymphoid

ANTIBODIES	FLUOROPHORE	CLONE	SOURSE	IDENTIFIER
Anti-CD45.1	PE	A20	BioLegend	Cat# 110707
Anti-CD45.2	PerCP-Cy5.5	104	BioLegend	Cat# 109827
Anti-CD11b	AF700	M1/70	BioLegend	Cat# 101222
Anti-CD19	APC-Cy7	6D5	BioLegend	Cat# 115529
Anti-CD3	PE/Dazzle594	145-2C11	BioLegend	Cat# 100347
Live dead	Zombie violet		BioLegend	Cat# 423113

Peripheral blood 2

ANTIBODIES	FLUOROPHORE	CLONE	SOURSE	IDENTIFIER
Anti-CD115	PE-Cy7	AFS98	invitrogen	25-1152-80
Anti-Ly6C	APC	HK1.4	BioLegend	Cat# 128015
Anti-Ly6G	PerCP-Cy5.5	1A8	BioLegend	Cat# 127616
Anti-B220	APC-Cy7	RA3-6B2	BioLegend	Cat# 103224
Anti-CD4	FITC	RM4-5	BioLegend	Cat# 100510
Anti-CD8a	BV510	SK1	BioLegend	Cat# 100751
Anti-CD3	BV711	17A2	BD Biosciences	Cat# 740739

Table. S2 Flow cytometry antibodies used in this study
