# Supplementary material for: Tet2-driven clonal hematopoiesis drives aortic aneurysm via macrophage-to-osteoclast–like differentiation
Source: J Clin Invest. 2026 Feb 25;136(8):e198708. doi: 10.1172/JCI198708 (PMC13078872; doi:10.1172/JCI198708)
Supplement: Unedited blot and gel images [file jci-136-198708-s023.pdf]

Unedited blot

Full unedited gel for Figure 4E

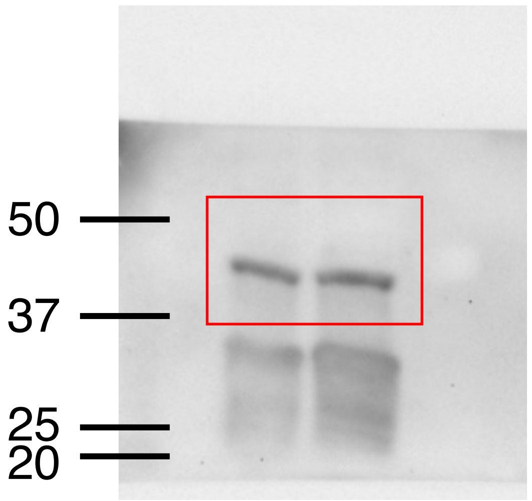

β-Actin (13E5) rabbit monoclonal antibody

(Cat# 4970, Cell Signaling Technology)

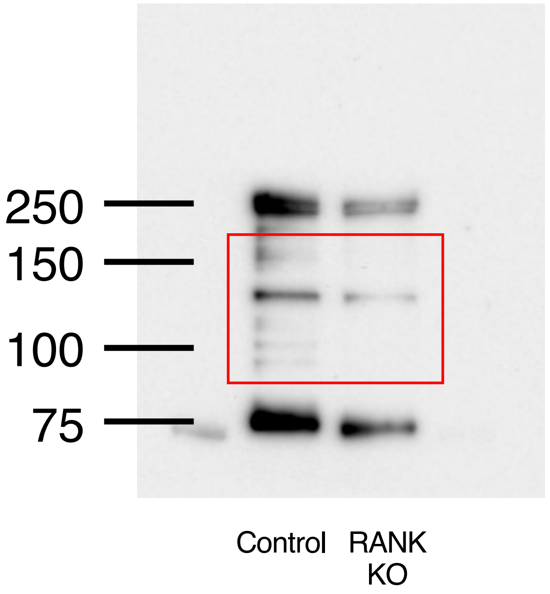

anti-RANK (EPR26196-15) rabbit monoclonal antibody

(Cat# ab305233, Abcam).
